# A photoswitchable [2]catenane receptor†

**DOI:** 10.1039/d4cc05934d

**Published:** 2025-01-15

**Authors:** Jorn de Jong, Maxime A. Siegler, Sander J. Wezenberg

**Affiliations:** a Leiden Institute of Chemistry, Leiden University Einsteinweg 55 2333 CC Leiden The Netherlands s.j.wezenberg@lic.leidenuniv.nl; b Department of Chemistry, Johns Hopkins University 3400 N. Charles St. Baltimore MD 21218 USA

## Abstract

A [2]catenane-based receptor functionalized with stiff-stilbene can be reversibly switched with 340/385 nm light between its *Z*- and *E*-isomers, which leads to a considerable change in chloride binding affinity. Photoisomerization in the presence of chloride allows for *in situ* on demand guest uptake and release.

## Abstract 2

Photoisomerization of a [2]catenane-based receptor functionalized with stiff-stilbene allows for on demand uptake and release of chloride ions.

## Graphical abstract

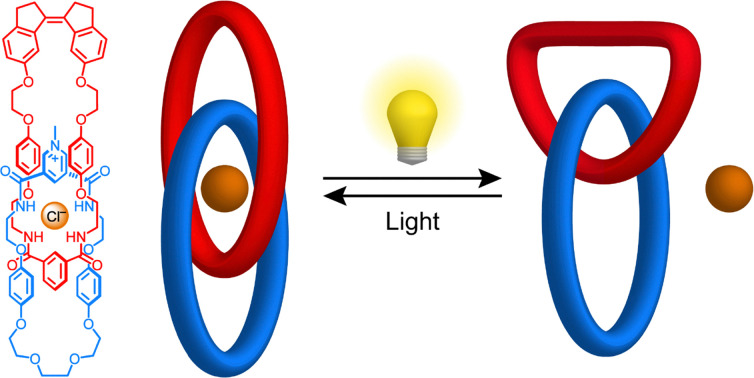

Owing to the important role of anionic species in various biological processes, during the past decades, a large number of artificial anion receptors have been developed.^1^ These receptors have found applications as extracting agents,^2^ analyte sensors,^3^ and membrane transporters.^4^ More recently, research efforts have been directed at dynamically modulating their binding affinity by using external stimuli,^5^ as it would allow controlled anion uptake and release in extractions and enable regulation of transport activity. The use of light as a stimulus towards this goal has proven particularly promising due to its high spatio-temporal precision, while no chemical waste is produced. The majority of light-responsive anion receptors developed up until now is based on a tweezer-type approach using a molecular photoswitch as the central core.^6,7^ Other strategies involve the incorporation of photoswitchable moieties into foldamers,^8^ or macrocycles,^9^ whereas the (un)blocking of hydrogen bond donating groups has also been used.^10^ In most designs, however, it remains challenging to achieve strong and selective binding, in particular in solvent media that are highly competitive with the anion–receptor interactions.

Among the various types of anion receptors developed to date, mechanically interlocked ones have shown a remarkably high binding affinity and selectivity, in particular for halide ions.^11,12^ Seminal work in this area by the group of Beer took advantage of directional hydrogen- and halogen-bonding interactions in a binding pocket formed between the two rings, or the ring and axle components in [2]catenanes and [2]rotaxanes, respectively.^12^ We identified such mechanically interlocked structures as ideal platforms for the development of light-switchable anion receptors. However, where stimuli-responsivity in mechanically interlocked molecules is increasingly used to control machine-like^13^ and catalytic functions,^14^ there are only a few examples in which anion guest binding was modulated, in these cases by addition of acid or alkali metal cations.^15^

Recently, we demonstrated control of anion-templated pseudo-rotaxane formation using a macrocycle that contained a stiff-stilbene (*i.e.* the fused five-membered ring analog of stilbene) photoswitch.^16^ This type of photoswitch has the advantage that both addressable states have high thermal stability and that it undergoes a large change in geometry upon *E*/*Z* isomerization.^17,18^ We planned to interlock this stiff-stilbene – as well as isophthalamide – containing macrocycle with another macrocycle bearing a pyridinium bis-amide anion-binding motif to afford a photoswitchable [2]catenane receptor (Scheme 1). Here, we report the synthesis and characterization of this receptor. The obtained isomer (*Z*)-1 strongly binds chloride, while irradiation with UV light to generate the *E*-isomer results in a significant decrease in affinity. In addition, the reversibility of the isomerization process is demonstrated, also in the presence of the anion, thus showing its successful uptake and release. To our knowledge, this work introduces the first example of a photoswitchable mechanically interlocked receptor and unlocks a new strategy to modulate substrate binding affinity.

**Scheme 1:**
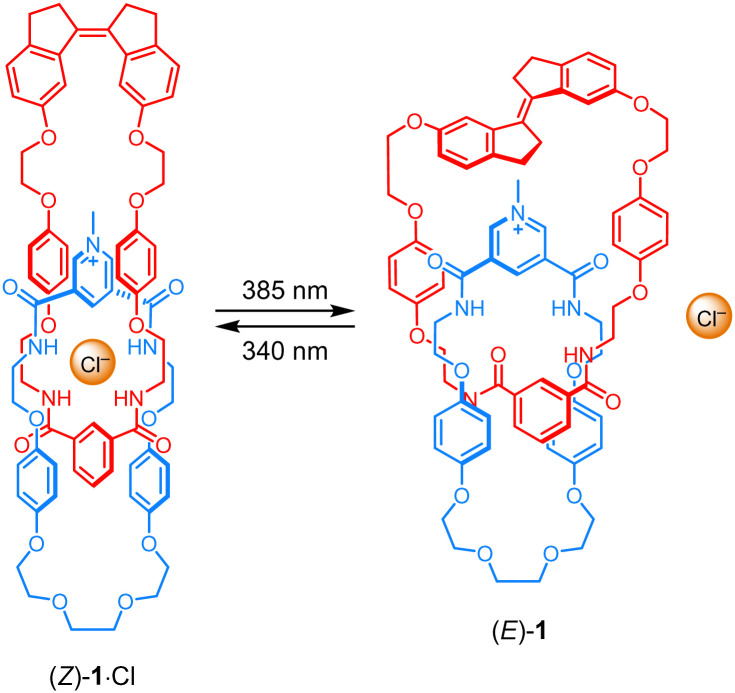
Light-induced chloride binding and release by [2]catenane receptor 1.

Catenane (*Z*)-1·Cl was prepared *via* an anion-templated clipping approach reported by the group of Beer,^19^ as shown in Scheme 2. Here, our recently reported diamine-functionalized stiff-stilbene (*Z*)-2^16^ was reacted with isophthaloyl chloride in the presence of pyridinium iodide macrocycle 3, which was prepared according to a procedure described by Beer and co-workers.^19^ The chloride ion could be removed by anion exchange using NH_4_PF_6_, yielding catenane (*Z*)-1·PF_6_ with the non-coordinating hexafluorophosphate counteranion.

**Scheme 2:**
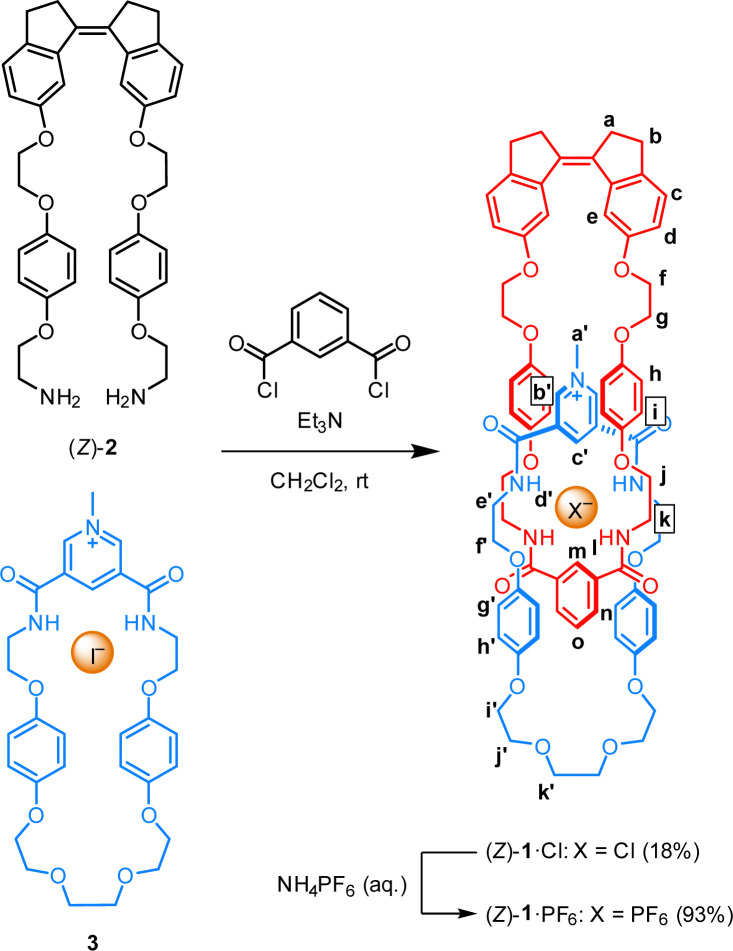
Synthesis of catenane (*Z*)-1·Cl and (*Z*)-1·PF_6_ by the clipping approach.

The mechanically interlocked nature was first confirmed by high resolution mass spectrometry (HRMS). The spectrum of (*Z*)-1·Cl featured signals whose *m*/*z* ratios were consistent with the positively charged interlocked rings without the chloride counterion (*m*/*z*: 1318.5601, *m*/*z* calcd: 1318.5595) as well as the doubly positively charged protonated species (*m*/*z*: 659.7833, *m*/*z* calcd: 659.7834) (see Fig. S14–S17 in the ESI†). Furthermore, the (^1^H,^1^H)-ROESY spectrum of (*Z*)-1·Cl in chloroform-*d* revealed multiple through-space interactions between protons belonging to the stiff-stilbene macrocycle and those of the pyridinium macrocycle, supporting that these components are interlocked (see Fig. S6 in the ESI†). For example, a cross-peak was observed between the pyridinium methyl proton H_a′_ and the stiff-stilbene proton H_e_ (see Scheme 2 for the lettering assignment). In addition, ROE contacts were found between the hydroquinone and methylene protons located around the chloride binding site of both components (*i.e.* H_i_ with H_e′_ and H_f′_; H_h′_ and H_g′_ with H_j_ and H_k_).

Conclusive evidence for catenane formation came from single-crystal X-ray crystallographic analysis. Suitable single crystals were obtained by slow evaporation of a solution of (*Z*)-1·Cl in acetonitrile/dichloromethane. The solid-state structure shows the two interlocked macrocycles coordinated to the chloride anion, which has a distorted tetrahedral geometry (see Fig. 1 and Fig. S33 in the ESI†). Each macrocycle is involved in two amide N(H)⋯Cl hydrogen bonds [distances between 3.408(2) and 3.4698(19) Å], as well as an additional C(H)⋯Cl interaction [distances of 3.519(2) and 3.622(3) Å]. The positively charged ring of the pyridinium macrocycle is located in between the two electron-rich hydroquinone groups of the stiff-stilbene-containing macrocycle as a result of π–π stacking interactions. Furthermore, the pyridinium methyl group is oriented towards the ethylene glycol moieties of the latter macrocycle. Similar observations were described for structurally related anion-templated interlocked and interpenetrated complexes.^16,19^ Finally, it should be noted that (*Z*)-stiff-stilbene can adopt enantiomeric helical conformations,^17^ and here, both (*P*)–(*Z*)-1·Cl and (*M*)–(*Z*)-1·Cl were found in the crystal lattice (see Fig. S34 in the ESI†).

**Fig. 1:**
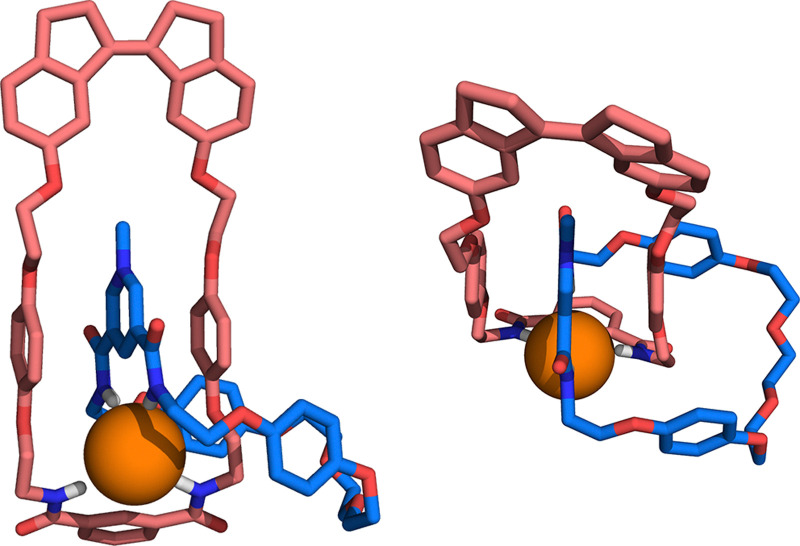
Front- and top-views of catenane (*Z*)-1·Cl as found in the crystal structure, shown in a capped stick representation. Disorder in the oligo-ethylene glycol part of the pyridinium macrocycle has been omitted for clarity.

Photoisomerization studies were performed first using the PF_6_^−^ salt in DMSO (for studies with the Cl^−^ complex, see below). The UV-vis spectrum of a solution of (*Z*)-1·PF_6_ showed two absorption maxima at *λ* = 350 nm and 362 nm (Fig. 2). Upon irradiation with 385 nm light, the maxima shifted to shorter wavelengths, *i.e. λ* = 344 nm and 361 nm, and the overall absorption increased, which is indicative of *Z* → *E* isomerization.^17^ Subsequent irradiation with 340 nm light led to opposite spectral changes, illustrative of isomerization back to the *Z*-isomer. The sample was irradiated until the photostationary states (PSS) had been reached and, during the isomerization process, an isosbestic point was maintained at *λ* = 366 nm, illustrating its unimolecular nature (Fig. S18 and S19 in the ESI†). Importantly, the 385/340 nm irradiation cycle could be repeated multiple times without significant signs of fatigue (Fig. 2, inset).

**Fig. 2:**
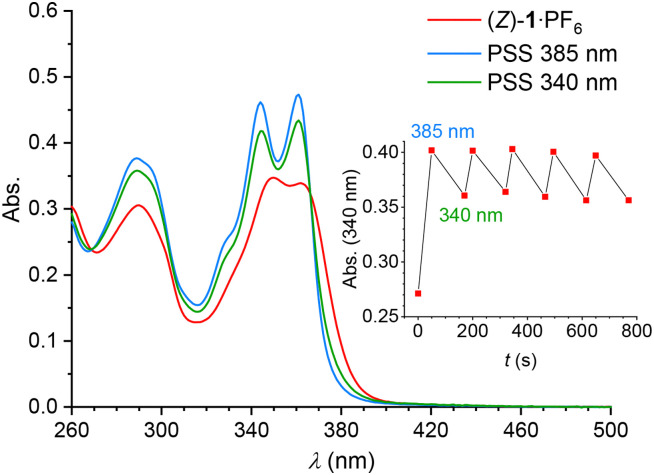
UV-vis spectral changes of (*Z*)-1·PF_6_ upon consecutive irradiation with 385 nm and 340 nm light (*c* = 2.0 × 10^−5^ M in dry and degassed DMSO) and change in absorption at 340 nm during 385/340 nm irradiation cycles (inset).

To determine the (*E*/*Z*)-ratios at the photostationary states, the same process was additionally studied by ^1^H NMR spectroscopy in DMSO-*d*_6_. Irradiation of a solution of (*Z*)-1·PF_6_ with 385 nm light resulted in the appearance of a new set of signals (Fig. S20–S22 in the ESI†), which could be assigned to (*E*)-1·PF_6_. For example, the signals of pyridinium protons H_d′_, H_b′_ and H_c′_, which were originally located at *δ* = 9.21, 9.17 and 8.95 ppm, now appeared at *δ* = 9.33, 9.46 and 9.07 ppm, respectively. Further, isophthalamide proton signals H_l_ and H_m_ shifted from *δ* = 8.43 and 8.38 ppm to *δ* = 8.55 and 8.18 ppm, respectively, and the chemical shift of aromatic stiff-stilbene proton H_e_ changed from *δ* = 7.48 ppm to *δ* = 7.12 ppm. Subsequent irradiation with 340 nm light partially reverted these ^1^H NMR spectral changes, confirming the regeneration of the *Z*-isomer. By ^1^H NMR signal integration PSS_340_ and PSS_385_ (*E*/*Z*)-ratios of 62 : 38 and 93 : 7 were calculated, respectively. These ratios are in line with values reported earlier for other cyclized stiff-stilbene photoswitches.^9*d*,18^

Chloride binding studies with both isomers of 1·PF_6_ were then carried out using ^1^H NMR spectroscopy in DMSO-*d*_6_, which is commonly used as a polar aprotic solvent for anion titrations.^1^ Stepwise addition of tetrabutylammonium chloride (NBu_4_Cl) to (*Z*)-1·PF_6_ led to significant shifting of the signals of most protons, in particular those involved in chloride coordination, indicating that the (*Z*)-1·Cl complex formed. That is, amide proton signals H_d′_ and H_l_ experienced an upfield (Δ*δ* = 0.18 ppm) and downfield shift (Δ*δ* = 0.13 ppm), respectively, as a result of hydrogen bonding to the chloride anion (Fig. S23 and S24 in the ESI†). Besides, the two signals of aromatic protons that are in close proximity to the binding site, H_c′_ and H_m_, shifted downfield (Δ*δ* = 0.15 ppm and 0.34 ppm, respectively). These spectral changes were fitted to a 1 : 1 binding equilibrium using HypNMR,^20^ giving an association constant (*K*_a,*Z*_) of 6.0 × 10^2^ M^−1^. A similar titration was performed with (*E*)-1·PF_6_, which was prepared by 385 nm irradiation of (*Z*)-1·PF_6_ (Fig. S25 and S26 in the ESI†). This time, the spectral changes observed upon the addition of NBu_4_Cl were smaller, and fitting the data to a 1 : 1 binding model afforded a stability constant (*K*_a,*E*_) of 1.3 × 10^2^ M^−1^, which is almost 5 times smaller than that obtained for the *Z*-isomer.

While we initially opted for DMSO as the solvent for our studies, titrations with earlier reported mechanically interlocked anion receptors were performed in chloroform-d/methanol-d_4_ (1 : 1, *v*/*v*). For comparison, we additionally determined the stability constants of the chloride complex in this solvent mixture.^12*a*,*b*,19^ Now, addition of NBu_4_Cl to (*Z*)-1·PF_6_ induced more pronounced spectral changes than in DMSO-*d*_6_ (Fig. S27 and S28 in the ESI†), in particular for proton signals H_b′_, H_c′_, and H_m_, which all shifted downfield (Δ*δ* = 0.13 ppm, 0.39 ppm, and 0.66 ppm, respectively). The association constant was found to be considerably larger in this case (*K*_a,*Z*_ = 3.0 × 10^3^ M^−1^) and is comparable to the constants determined for structurally related catenated anion receptors.^12^ Remarkably, the affinity of (*E*)-1·PF_6_ for chloride in this solvent mixture was nearly 12 times lower (*K*_a,*E*_ = 2.6 × 10^2^ M^−1^) as compared to (*Z*)-1·PF_6_ (Fig. S29–S31 in the ESI†), whereas both the *K*_a,*Z*_ and *K*_a,*E*_ values were larger than what was determined in DMSO-*d*_6_. The large decrease in binding strength upon photoisomerization from the *Z*- to *E*-isomer is most likely caused by increased steric congestion at the anion binding site, *viz.* the *Z* → *E* geometry change of the photoswitchable macrocycle leads to a reduced distance between its stiff-stilbene and isophthalamide moieties, leading to a smaller void space, in which the pyridinium bis-amide motif of the secondary macrocycle needs to squeeze in to form a suitable chloride binding pocket.

Lastly, photoisomerization was performed with the chloride-bound complex *in situ*, and concomitant uptake and release of the anion was followed using ^1^H NMR spectroscopy. First a solution of (*Z*)-1·PF_6_ in DMSO-*d*_6_ (1.0 mM) was mixed with 15 equivalents of NBu_4_Cl (Fig. 3 and Fig. S32 in the ESI†). Based on the chemical shifts of the protons located in the binding site (H_c′_, H_d′_, H_l_ and H_m_), it was calculated that 93% of the *Z*-isomer was associated with chloride, in agreement with the determined association constant *K*_a,*Z*_ of 6.0 × 10^2^ M^−1^ (*vide supra*). Irradiation of this solution with 385 nm light gave a mixture with an (*E*/*Z*)-ratio of 89 : 11 at the PSS, where now only 63% of the *E*-isomer was complexed with chloride, again in line with the (lower) *K*_a,*E*_ value of 1.3 × 10^2^ M^−1^. This means that overall nearly 30% of the originally bound chloride was released. Subsequent irradiation with 340 nm light gave a mixture with an (*E*/*Z*)-ratio of 58 : 42 at the PSS and thus, led to a partial reuptake of chloride.

**Fig. 3:**
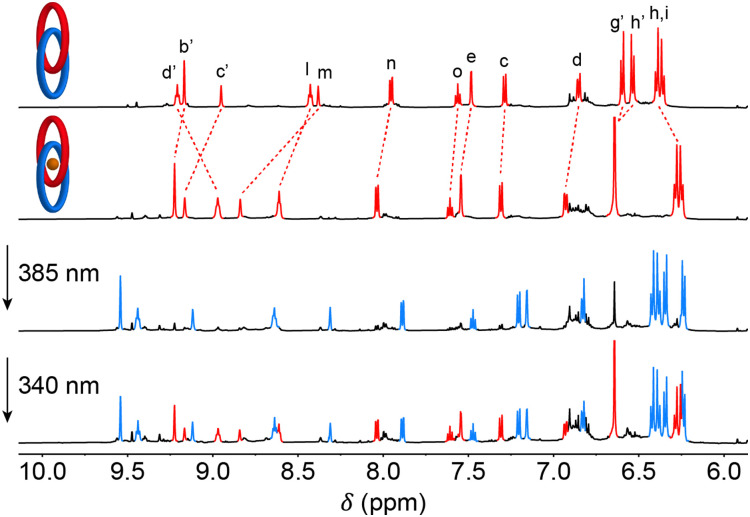
^1^H NMR spectral changes upon addition of 15 equivalents of NBu_4_Cl to (*Z*)-1·PF_6_ (1.0 mM in DMSO-*d*_6_), followed by irradiation with 385 nm and 340 nm light. The ^1^H NMR signals of (*Z*)-1 and (*E*)-1 are highlighted in red and blue, respectively.

In summary, we have presented a light-switchable [2]catenane receptor for chloride. Reversible isomerization between its *Z*- and *E*-isomers could be induced by 340/385 nm light and was shown to cause a considerable change in binding affinity. Importantly, this isomerization in the presence of chloride allowed its controlled uptake and release. Our work unlocks an approach to effectively modulate anion binding strength in mechanically interlocked receptors, which will prove useful because of their strong binding and high levels of anion selectivity.^11,12^ Further, the possibility to alter the stability of these anion-templated structures on demand by using light facilitates the development of new types of molecular machines,^13^ including anion pumps, which we aim to demonstrate in the future.

Financial support from the European Research Council (starting grant no. 802830 to S. J. W.) is gratefully acknowledged. We thank Dr Karthick B. Sai Sankar Gupta and Dr Maria I. Nardella for their help with NMR experiments and Hans van den Elst for performing HRMS analyses.

## Data availability

The data supporting this article have been included as part of the ESI.† Crystallographic data for (*Z*)-1·Cl has been deposited at the CCDC under 2400475.†

## Conflicts of interest

There are no conflicts to declare.

## Supplementary Material

CC-061-D4CC05934D-s001

CC-061-D4CC05934D-s002
