# Supplementary material for: A photoswitchable [2]catenane receptor
Source: Chem Commun (Camb). 2025 Jan 15;61(12):2548–51. doi: 10.1039/d4cc05934d (PMC11734587; doi:10.1039/d4cc05934d)
Supplement: CC-061-D4CC05934D-s001 [file CC-061-D4CC05934D-s001.pdf]

Supplementary Information (SI) for ChemComm.
This journal is © The Royal Society of Chemistry 2024

ELECTRONIC SUPPLEMENTARY INFORMATION (ESI)

A photoswitchable [2]catenane receptor

Jorn de Jong,[a] Maxime A. Siegler,[b] and Sander J. Wezenberg*[a]

[a] *Leiden Institute of Chemistry, Leiden University, Einsteinweg 55, 2333 CC Leiden, The Netherlands*

[b] *Department of Chemistry, Johns Hopkins University, 3400 N. Charles St., Baltimore, MD, 21218, United States*

Email: s.j.wezenberg@lic.leidenuniv.nl

Table of Contents

Experimental section S1

NMR spectra of title compounds S4

HRMS spectra of title compounds S12

UV-Vis irradiation experiments S14

^{1}H NMR irradiation experiments S15

^{1}H NMR titration experiments S17

^{1}H NMR irradiation in presence of chloride S23

Single crystal X-ray crystallography S25

References S28

Experimental section

General methods and materials:

CH_2Cl_2 was dried using a Pure Solve 400 solvent purification system from Innovative Technology. Dry Et_3N was purchased from Sigma Aldrich, and DMSO-d_6, $CDCl_3$ and CD_3OD were purchased from Eurisotop. Where indicated, solvents were degassed by purging with N_2 for 30 min unless noted otherwise. Diamine-functionalized stiff-stilbene (*Z*)-**2** and macrocycle **3** were prepared according to procedures reported in the literature.[1,2] All other chemicals were commercial products and were used without further purification. Column chromatography was performed using silica gel (SiO_2) purchased from Screening Devices BV (pore diameter 55-70 Å, surface area 500 m^2g^{-1}) and thin-layer chromatography (TLC) was carried out on aluminum sheets coated with silica 60 F254 obtained from Merck. Compounds were visualized with UV light (254 nm). ^1H, 2D (^1H-^{13}C), ^{19}F and ^{31}P NMR spectra were recorded on Bruker AV 400WB and Bruker AV 600 instruments at the indicated temperature. Chemical shifts (δ) are denoted in parts per million (ppm) relative to residual protiated solvent (DMSO-d_6: for ^1H detection, δ = 2.50 ppm; for ^{13}C detection, δ = 39.52 ppm; $CDCl_3$: for ^1H detection, δ = 7.26 ppm; for ^{13}C detection, δ = 77.16 ppm; CD_3OD: for ^1H detection, δ = 3.31 ppm). The splitting pattern of peaks is designated as follows: s (singlet), d (doublet), t (triplet), q (quartet), dd (doublet of doublets), sept (septet), m (multiplet). IR spectra were recorded on a Perkin Elmer Spectrum Two FT-IR spectrometer. The intensity of bands (ν = cm^{-1}) is assigned as follows: s (strong), m (medium), w (weak), very w (very weak), br (broad), and sh (shoulder). High-resolution mass spectrometry (ESI-MS) was performed on a Thermo Scientific Q Exactive HF spectrometer with ESI ionization. UV-Vis spectra were recorded on an Agilent Cary 8454 spectrometer in a 1 cm quartz cuvette. Irradiation of UV-Vis and NMR samples was carried out using Thorlabs model M340F3 (1.06 mW), M340L4 (60 mW) and M385F1 (10.7 mW) LEDs, positioned at a distance of 1 cm to the sample.

Catenane (*Z*)-1·Cl:

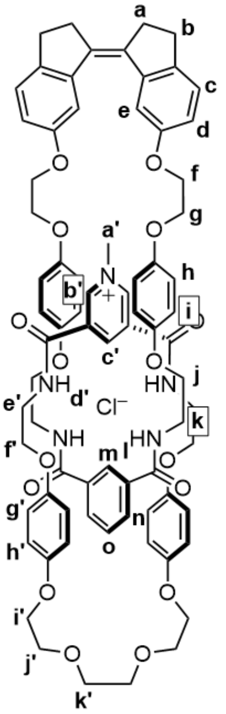

Stiff-stilbene diamine (*Z*)-**2** (18 mg, 29 µmol) and pyridinium iodide macrocycle **3** (20 mg, 29 µmol, 1 eq.) were dissolved in dry CH_2Cl_2 (6 mL) in an oven-dried round-bottom flask under a nitrogen atmosphere. After stirring for 5 min at rt, Et_3N (10 µL, 72 µmol, 2.5 eq.) was added, which was immediately followed by the dropwise addition of a 50 mM solution of isophthaloyl chloride in dry CH_2Cl_2 (0.60 mL, 30 µmol, 1 eq.). The reaction mixture was stirred at rt for 1.5 h and subsequently diluted with CH_2Cl_2 to reach a total volume of 15 mL. It was then washed with 1 M HCl aq. (2 × 7.5 mL) and water (2 × 7.5 mL), dried over anhydrous Na_2SO_4, and concentrated. Purification by column chromatography (SiO_2; CH_2Cl_2/MeOH 95:5 to 90:10) gave catenane (*Z*)-**1**·Cl as a light yellow solid (7 mg, 18%); R_f = 0.54 (SiO_2; CH_2Cl_2/MeOH 90:10); ^{1}H NMR (600 MHz, $CDCl_3$; assignments are based on COSY, NOESY and ROESY NMR spectra) δ = 9.76 (s, 1H; $H_{c'}$), 9.12 (t, J = 1.6 Hz, 1H; H_m), 8.77 (s, 2H; $H_{b'}$), 8.66 (t, J = 4.4 Hz, 2H; $H_{d'}$), 8.52 (t, J = 3.9 Hz, 2H; H_l), 8.29 (dd, J = 7.8, 1.2 Hz, 2H; H_n), 7.63 (d, J = 2.3 Hz, 2H; H_e), 7.61 (t, J = 7.8 Hz, 1H; H_o), 7.30 (d, J = 8.3 Hz, 2H; H_c), 6.87 (dd, J = 8.3, 2.3 Hz, 2H; H_d), 6.75 (d, J = 9.1 Hz, 4H; $H_{h'}$), 6.66 (d, J = 9.1 Hz, 4H; $H_{g'}$), 6.34 (d, J = 8.9 Hz, 4H; H_i), 6.14 (d, J = 8.9 Hz, 4H; H_h), 4.71 (s, 3H; $H_{a'}$), 4.28 – 4.22 (m, 4H; H_f), 4.15 – 4.10 (m, 4H; $H_{i'}$), 4.06 (t, J = 4.5 Hz, 4H; $H_{f'}$), 3.99 – 3.94 (m, 4H; H_g), 3.89 (q, J = 4.4 Hz, 4H; $H_{e'}$), 3.78 – 3.73 (m, 4H; $H_{j'}$), 3.70 – 3.61 (m, 8H; H_j, $H_{k'}$), 3.41 (q, J = 3.9 Hz, 4H; H_k), 3.01 – 2.95 (m, 4H; H_b), 2.90 – 2.84 (m, 4H; H_a) ppm; ^{13}C NMR (151 MHz, $CDCl_3$; due to the low quantity that was isolated no ^{13}C NMR spectrum could be recorded and chemical shifts were therefore derived from (^{1}H,^{13}C)-HSQC and (^{1}H,^{13}C)-HMBC spectra) δ = 156.8, 152.7, 152.6, 151.6, 144.2, 142.3, 142.0, 136.4, 135.8, 133.6, 131.8, 129.0, 126.0, 124.2, 116.0, 114.9, 114.7, 114.6, 112.3, 111.9, 70.2, 69.4, 68.4, 68.2, 67.9, 66.1, 65.1, 49.6, 41.1, 39.9, 35.6, 29.7 ppm (4 signals were not visible due to low intensity); IR (ATR) ν = 3283 (br. w), 3066 (br. very w), 2923 (m), 2852 (w), 1729 (very w), 1679 (m, sh), 1660 (m), 1539 (w), 1508 (s), 1459 (w), 1400 (very w), 1368 (very w), 1291 (w), 1228 (s), 1109 (w), 1066 (m), 946 (w), 827 (w), 733 cm^{-1} (very w); HRMS (ESI) *m*/*z*: 1318.5601 ([M]$^+$, calcd for $C_{76}H_{80}N_5O_{16}^+$: 1318.5595); *m*/*z*: 659.7833 ([M+H]$^{2+}$, calcd for $C_{76}H_{81}N_5O_{16}^{2+}$: 659.7834).

Catenane (*Z*)-1·PF_6:

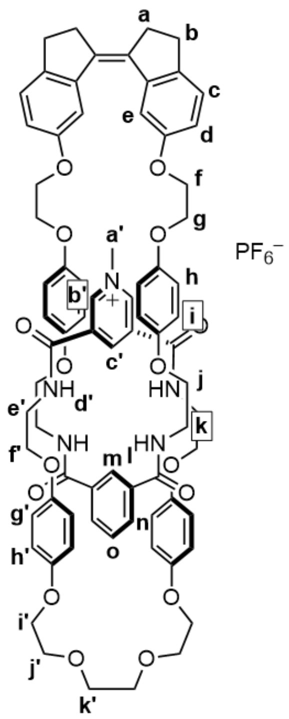

An aqueous 1 M NH_4PF_6 solution (5 mL) was added to catenane (*Z*)-**1**·Cl (5 mg, 4 µmol) in $CHCl_3$ (5 mL) and the biphasic mixture was stirred vigorously at rt for 1 h. The organic layer was separated, washed with water (2 × 5 mL), and concentrated to obtain (*Z*)-**1**·PF_6 as a light yellow solid (5 mg, 93%); ^{1}H NMR (600 MHz, DMSO-d_6; assignments are based on COSY and NOESY NMR spectra) δ = 9.20 (t, J = 5.2 Hz, 2H; $H_{d'}$), 9.17 (s, 2H; $H_{b'}$), 8.95 (s, 1H; $H_{c'}$), 8.43 (t, J = 5.2 Hz, 2H; H_l), 8.38 (s, 1H; H_m), 7.95 (dd, J = 7.7, 1.0 Hz, 2H; H_n), 7.56 (t, J = 7.7 Hz, 1H; H_o), 7.48 (d, J = 1.7 Hz, 2H; H_e), 7.29 (d, J = 8.3 Hz, 2H; H_c), 6.85 (dd, J = 8.3, 1.7 Hz, 2H; H_d), 6.60 (d, J = 9.1 Hz, 4H; $H_{g'}$), 6.54 (d, J = 9.1 Hz, 4H; $H_{h'}$), 6.40 (d, J = 9.1 Hz, 4H; H_h), 6.36 (d, J = 9.1 Hz, 4H; H_i), 4.37 (s, 3H; $H_{a'}$), 4.10 (t, J = 4.8 Hz, 4H; H_f), 3.91 (t, J = 4.8 Hz, 4H; H_g), 3.88 (t, J = 4.1 Hz, 4H; $H_{f'}$), 3.83 – 3.79 (m, 4H; $H_{i'}$), 3.74 (t, J = 4.7 Hz, 4H; H_j), 3.56 (q, J = 4.1 Hz, 4H; $H_{e'}$), 3.51 (q, J = 4.7 Hz, 4H; H_k), 3.49 – 3.46 (m, 4H; $H_{j'}$), 3.38 (s, 4H; $H_{k'}$), 2.93 – 2.87 (m, 4H; H_b), 2.84 – 2.77 (m, 4H; H_a) ppm; ^{13}C NMR (151 MHz, DMSO-d_6; due to the low quantity that was isolated no ^{13}C NMR spectrum could be recorded and chemical shifts were therefore derived from (^{1}H,^{13}C)-HSQC and (^{1}H,^{13}C)-HMBC spectra) δ = 166.4, 160.6 156.8, 152.3, 152.2 (2), 151.9, 145.7, 141.0, 140.9, 140.2, 135.3, 134.3, 132.7, 129.9, 128.3, 125.8, 125.6, 114.8, 114.6 (3), 112.9, 110.2, 69.6, 68.6, 66.9, 66.7, 66.1 (3), 48.1, 39.3, 38.9, 34.9, 29.0 ppm; ^{19}F NMR (376 MHz, DMSO-d_6) δ = –70.4 (d, J = 711 Hz) ppm; ^{31}P{^{1}H} NMR (162 MHz, DMSO-d_6) δ = –144.2 (sept, J = 711 Hz) ppm; IR (ATR) ν = 3314 (br. w), 3075 (br. very w), 2923 (m), 2852 (w), 1684 (m, sh), 1645 (m), 1543 (m), 1508 (s), 1483 (m, sh), 1459 (m), 1290 (m), 1226 (s), 1106 (w), 1065 (m), 943 (w), 845 (s), 738 cm^{-1} (w); HRMS (ESI) *m*/*z*: 1318.5596 ([M]$^+$, calcd for $C_{76}H_{80}N_5O_{16}^+$: 1318.5595); *m*/*z*: 659.7832 ([M+H]$^{2+}$, calcd for $C_{76}H_{81}N_5O_{16}^{2+}$: 659.7834).

NMR spectra of title compounds

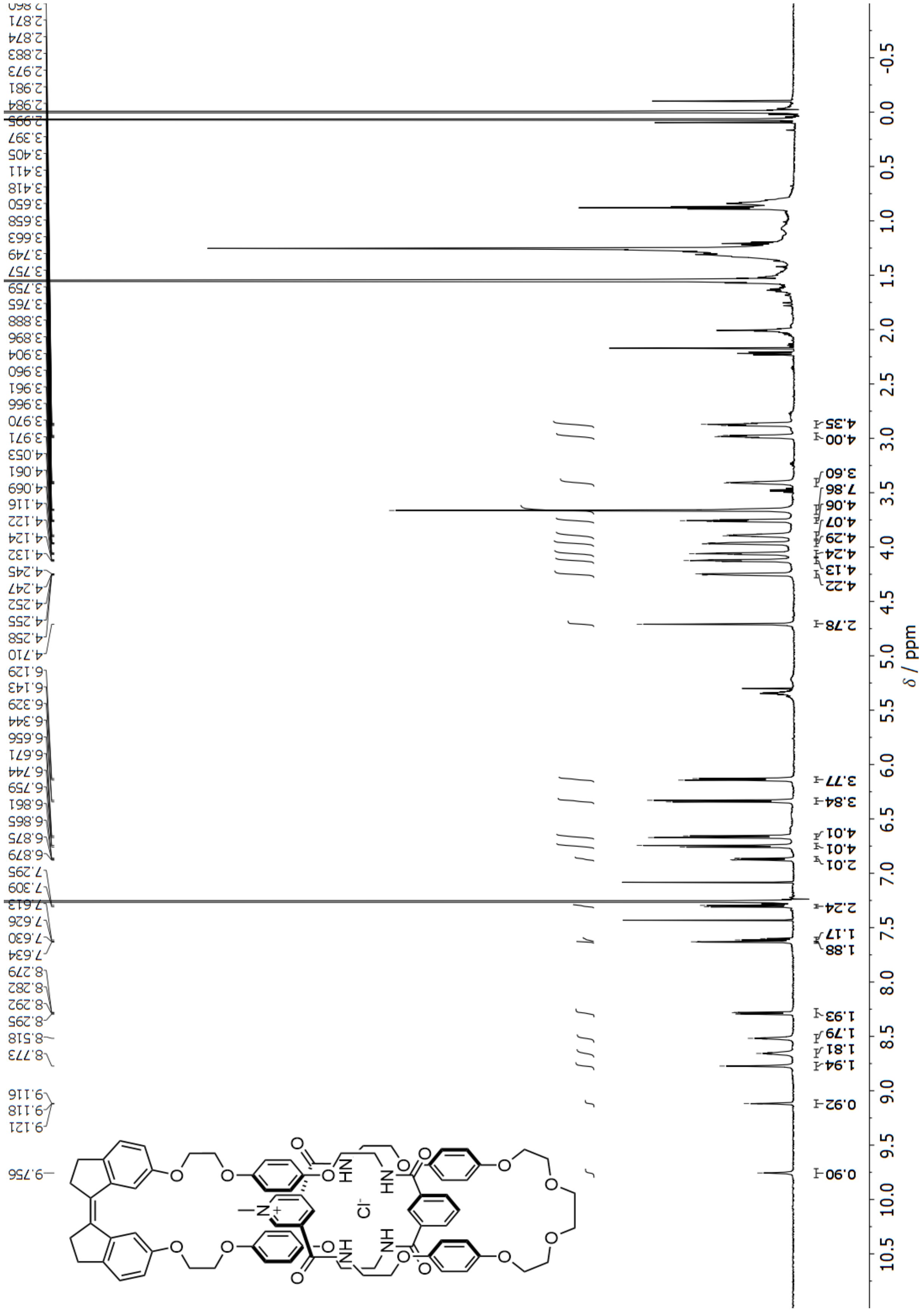

Figure S1. ^{1}H NMR spectrum (600 MHz, $CDCl_3$) of compound (*Z*)-**1**·Cl measured at 298 K.

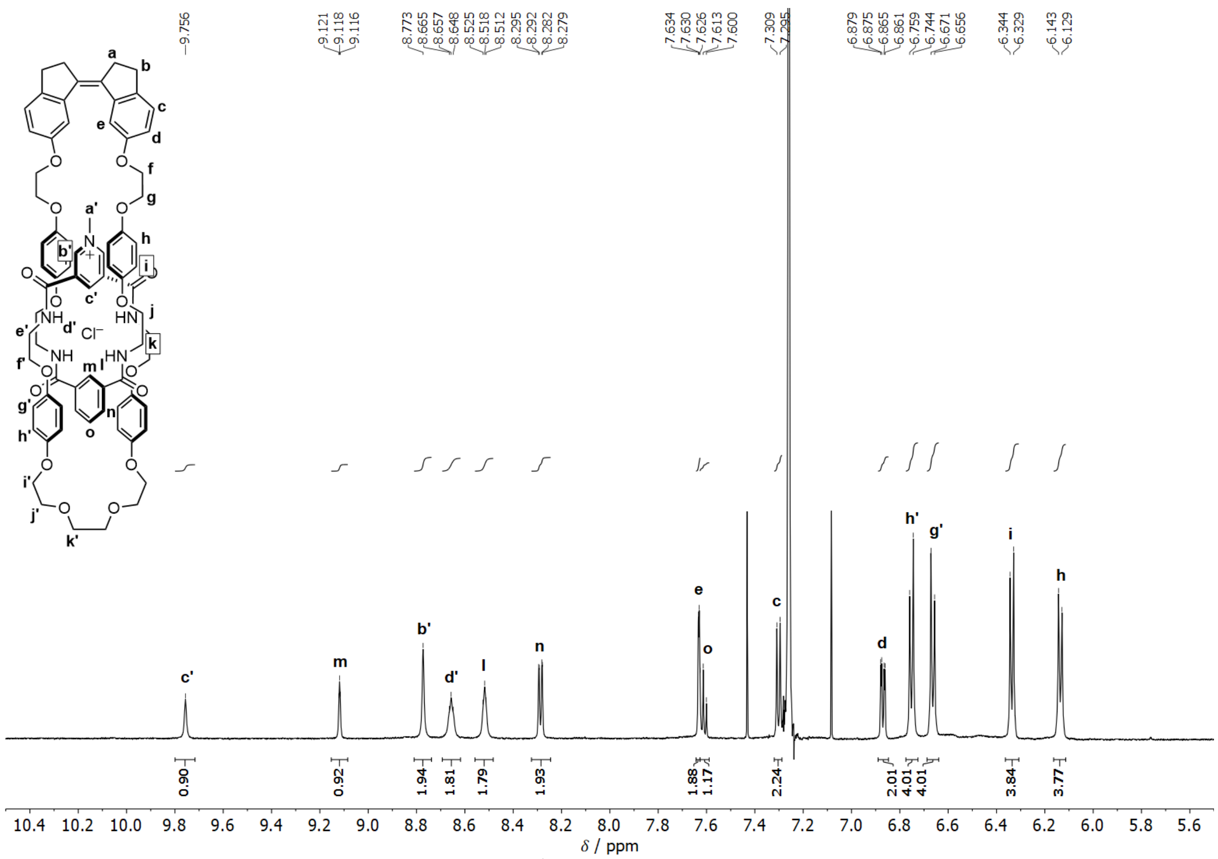

Figure S2. Selected downfield region in the ^{1}H NMR spectrum (600 MHz, $CDCl_3$) of compound (*Z*)-**1**·Cl measured at 298 K, including proton assignment.

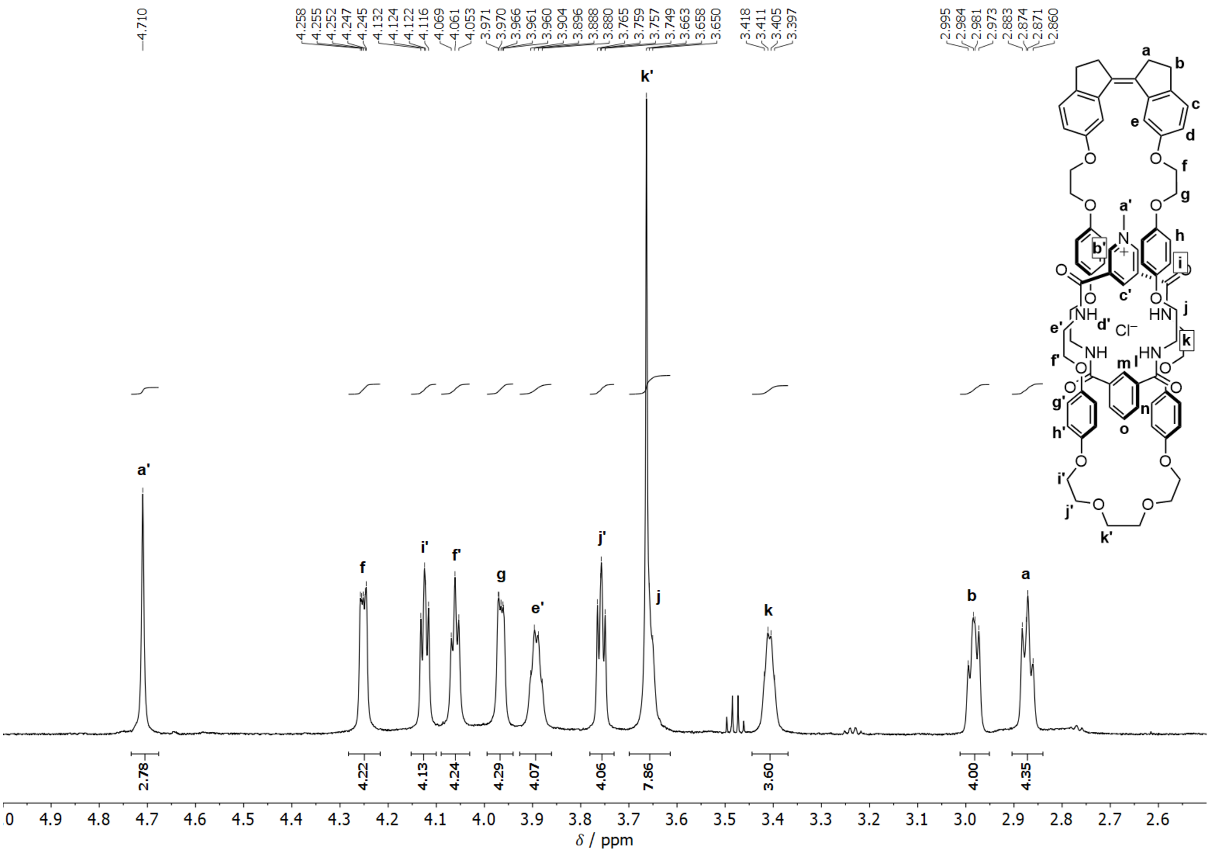

Figure S3. Selected upfield region in the ^{1}H NMR spectrum (600 MHz, $CDCl_3$) of compound (*Z*)-**1**·Cl measured at 298 K, including proton assignment.

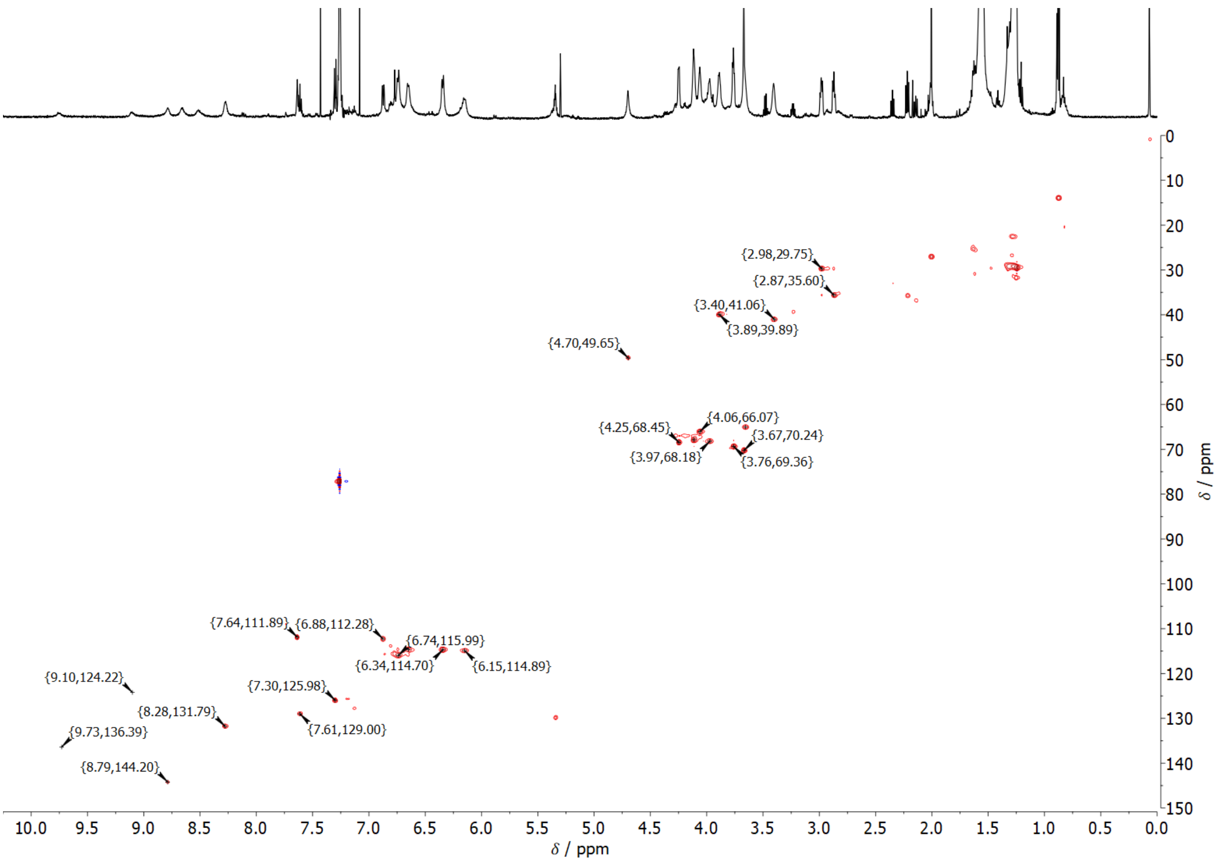

Figure S4. (^{1}H,^{13}C)-HSQC NMR spectrum (600/151 MHz, $CDCl_3$) of compound (*Z*)-**1**·Cl measured at 298 K.

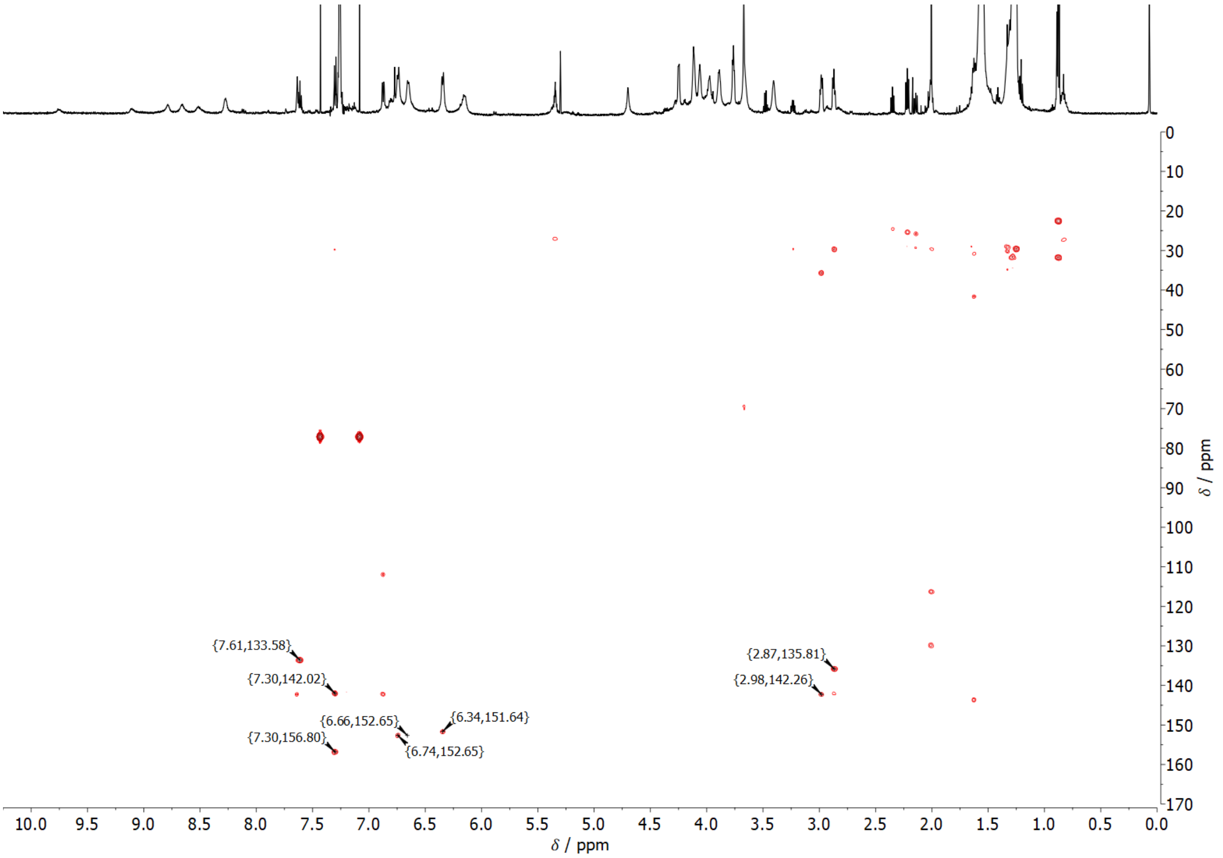

Figure S5. (^{1}H,^{13}C)-HMBC NMR spectrum (600/151 MHz, $CDCl_3$) of compound (*Z*)-**1**·Cl measured at 298 K.

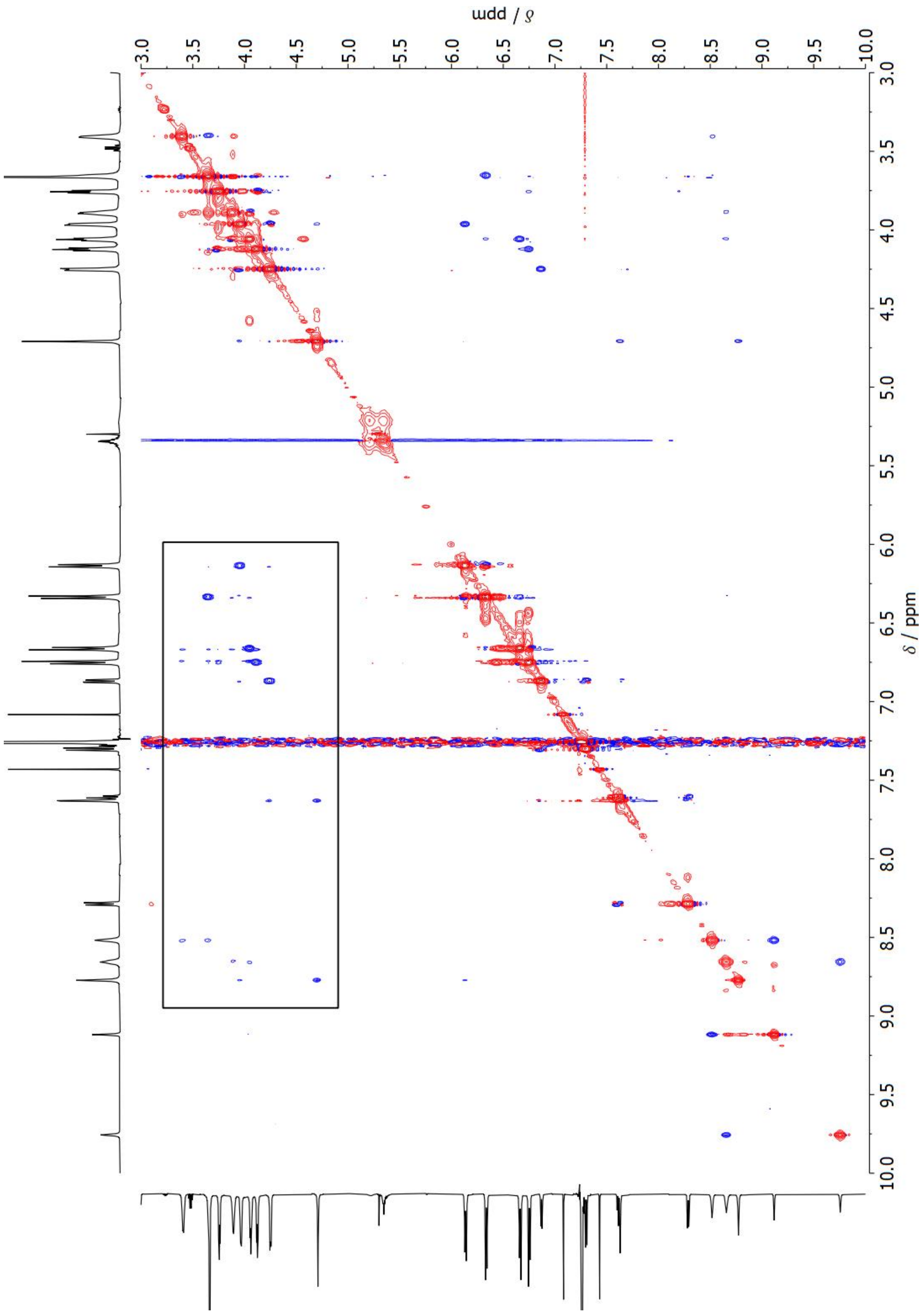

Figure S6. (^{1}H,^{1}H)-ROESY NMR spectrum (600 MHz, $CDCl_3$, τ_m = 200 ms) of compound (*Z*)-**1**·Cl measured at 298 K. The cross-peaks within the indicated area confirm the mechanically interlocked nature of catenane (*Z*)-**1**·Cl.

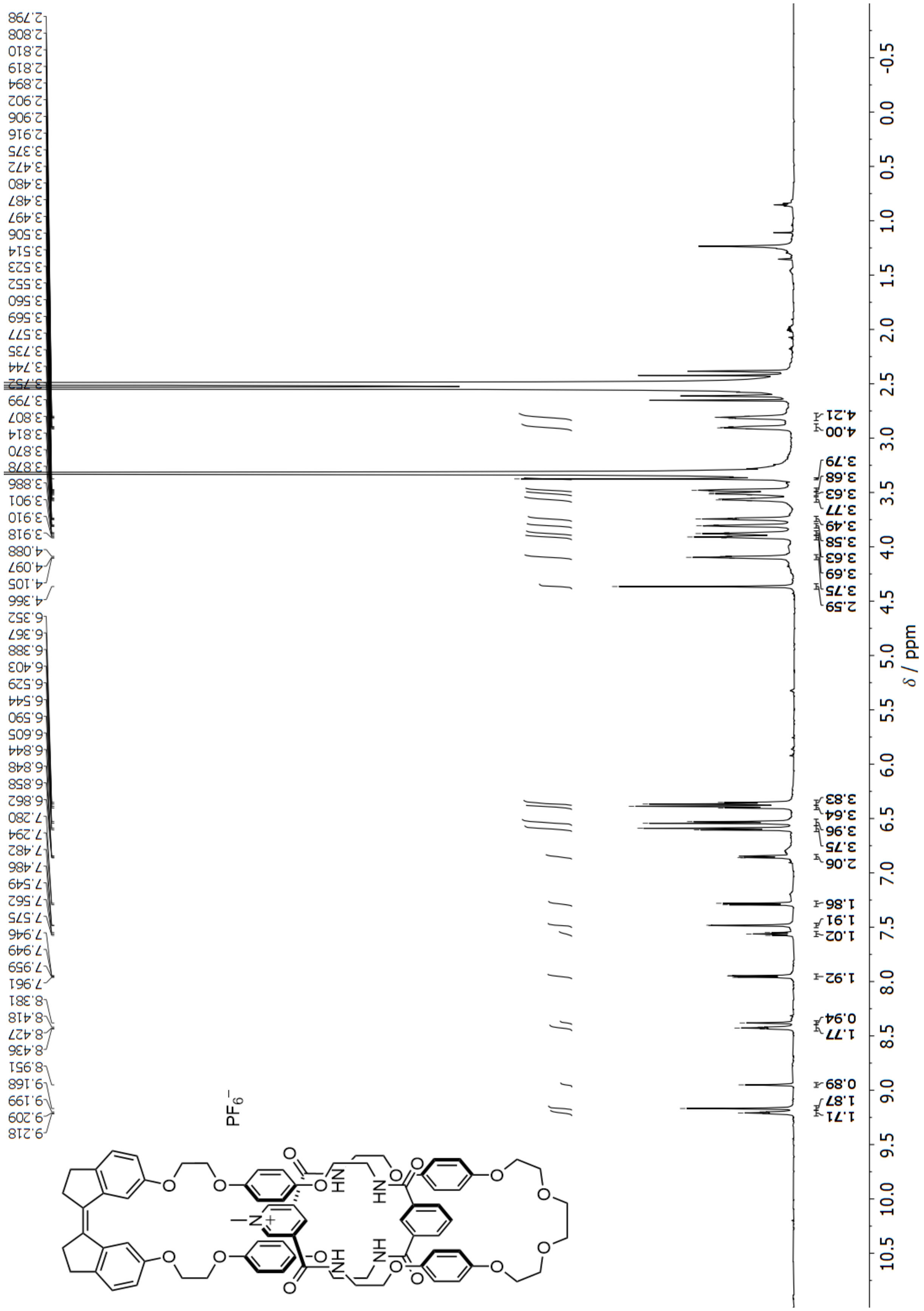

Figure S7. ^{1}H NMR spectrum (600 MHz, DMSO-d_6) of compound (*Z*)-**1**·PF_6 measured at 298 K.

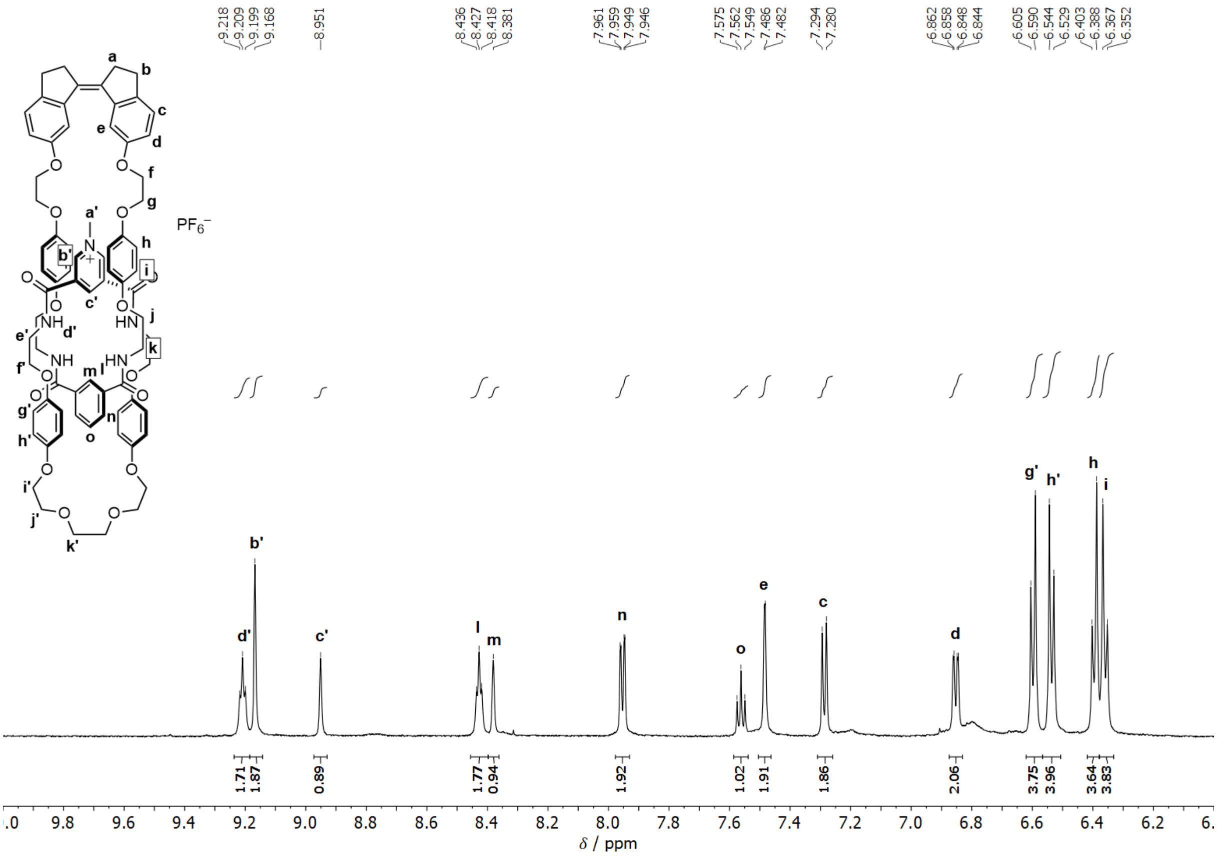

Figure S8. Selected downfield region in the ^{1}H NMR spectrum (600 MHz, DMSO-d_6) of compound (Z)-**1**·PF_6 measured at 298 K, including proton assignment.

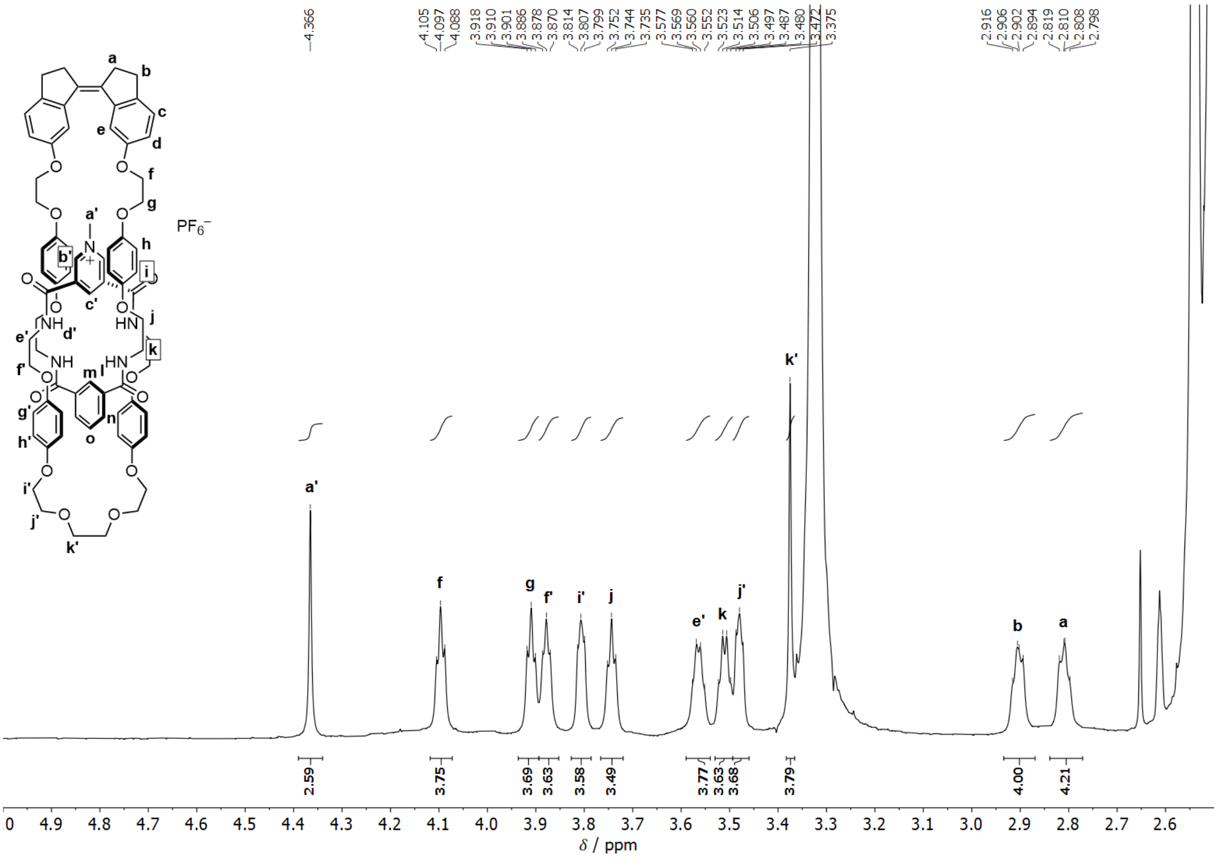

Figure S9. Selected upfield region in the ^{1}H NMR spectrum (600 MHz, DMSO-d_6) of compound (Z)-**1**·PF_6 measured at 298 K, including proton assignment.

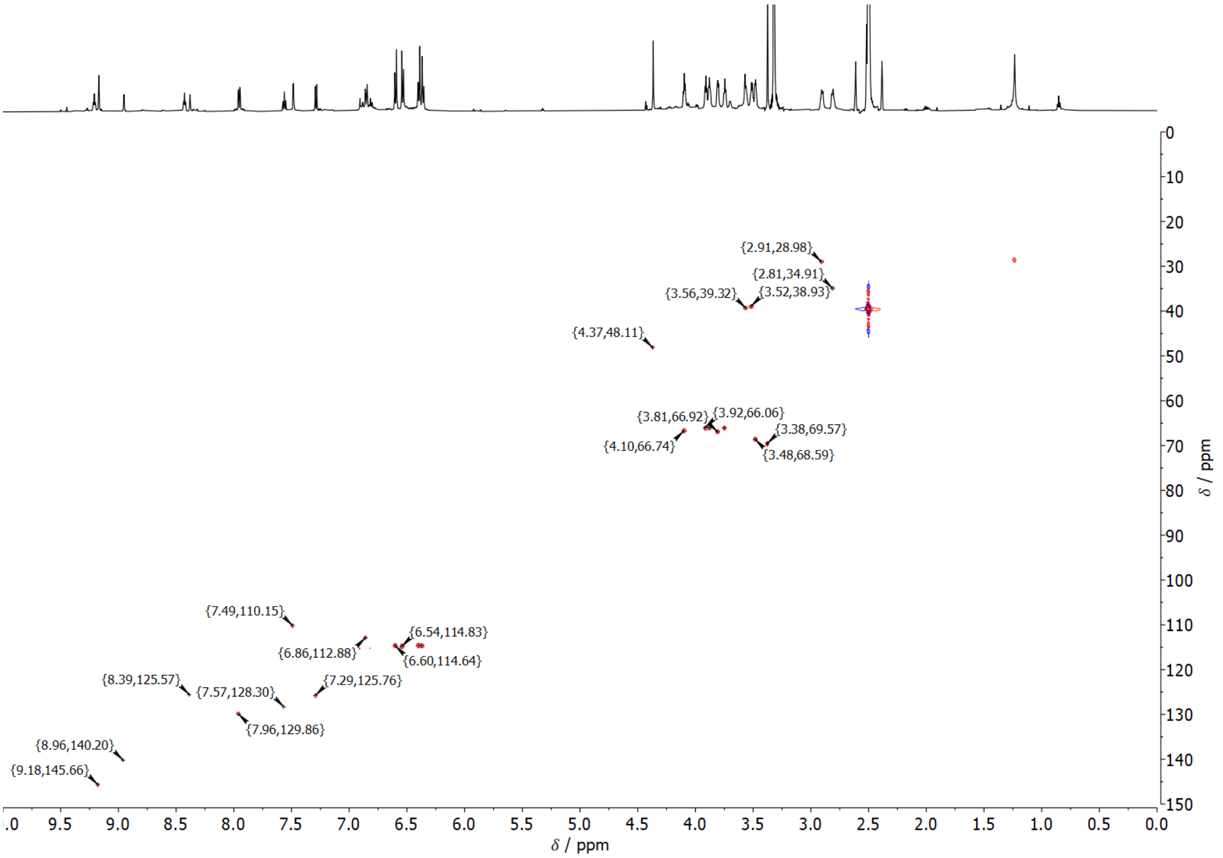

Figure S10. (^{1}H,^{13}C)-HSQC NMR spectrum (600/151 MHz, DMSO-d_6) of (*Z*)-**1**·PF_6 measured at 298 K.

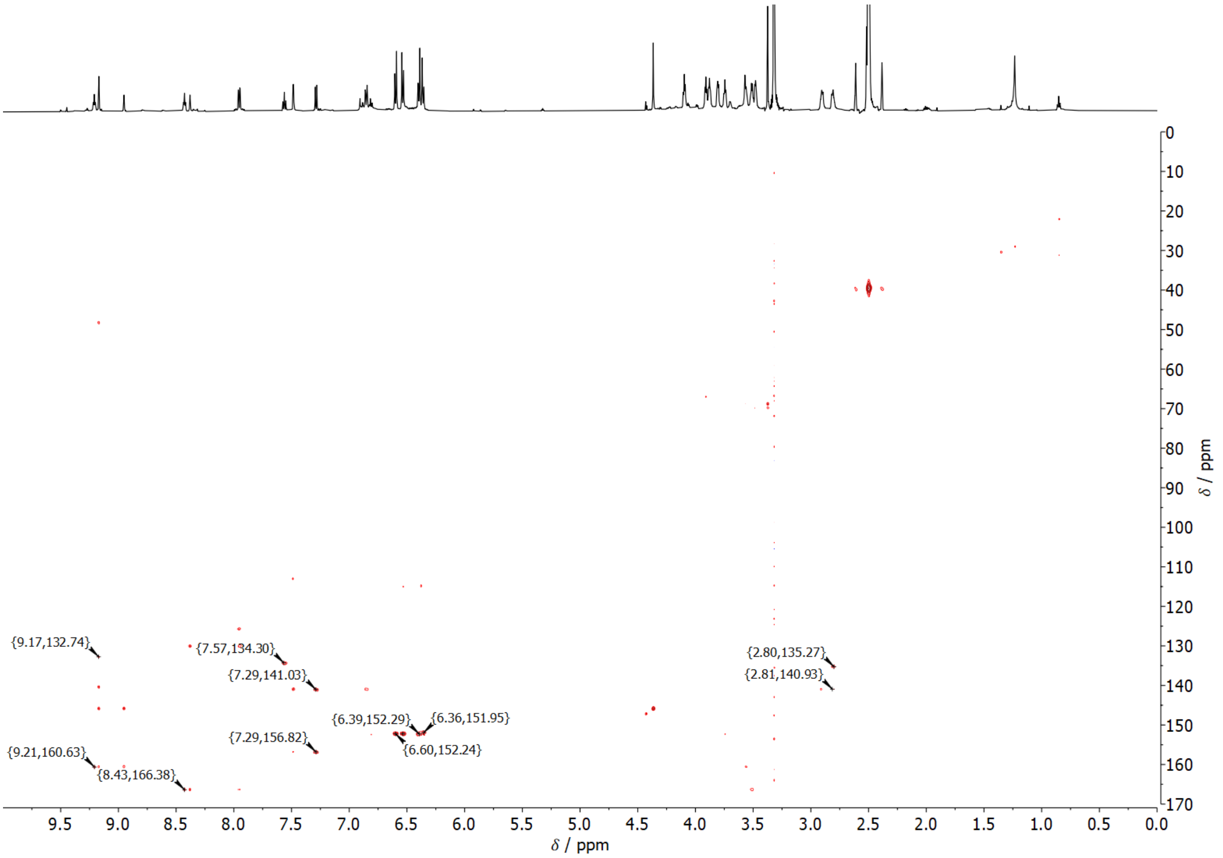

Figure S11. (^{1}H,^{13}C)-HMBC NMR spectrum (600/151 MHz, DMSO-d_6) of (*Z*)-**1**·PF_6 measured at 298 K.

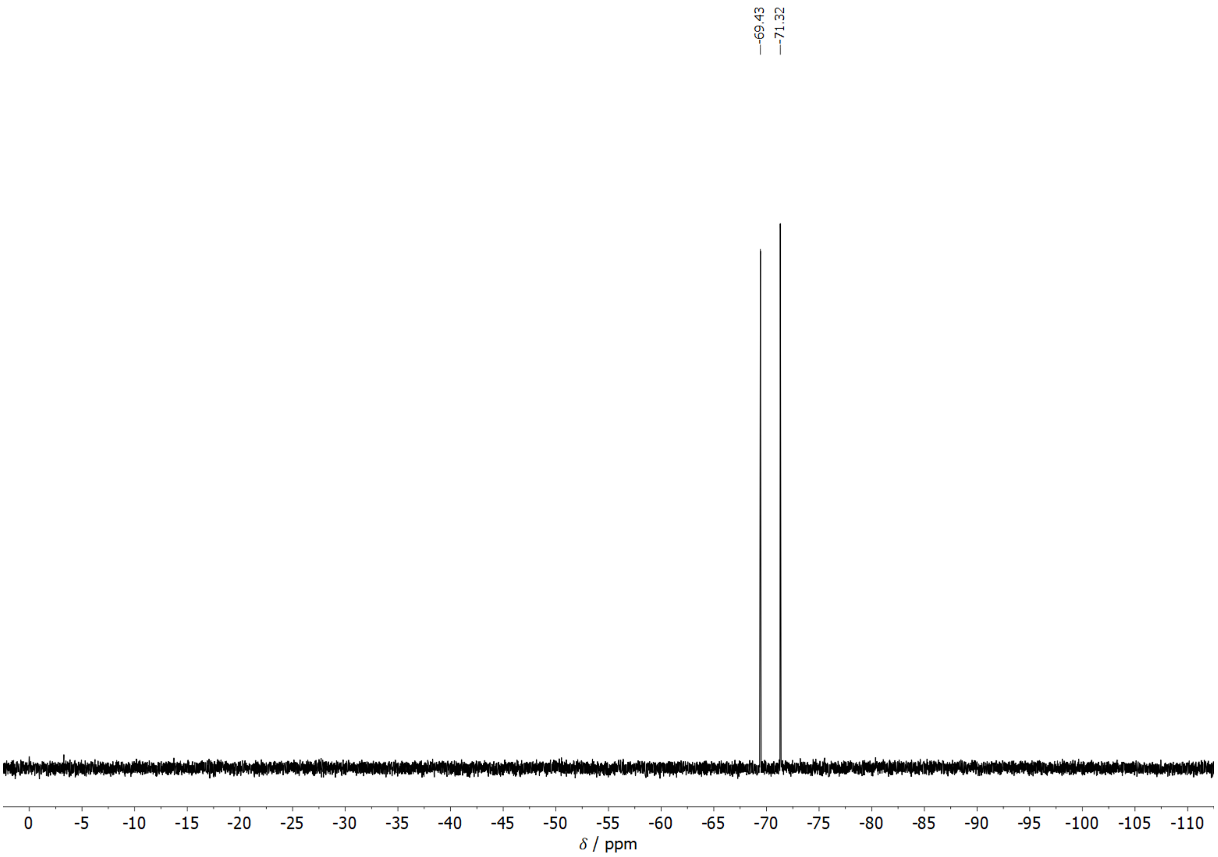

Figure S12. ^{19}F NMR spectrum (376 MHz, DMSO-d_6) of compound (*Z*)-**1**·PF_6 measured at 294 K.

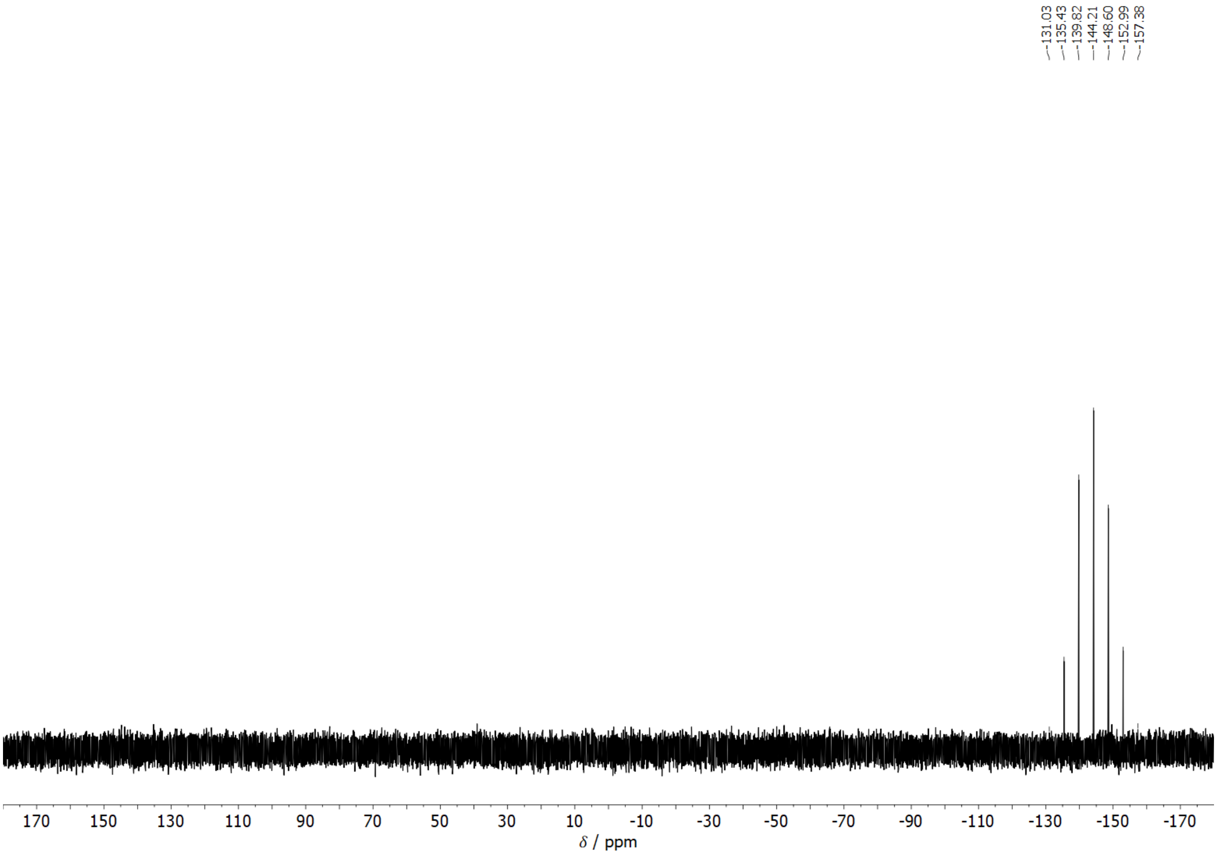

Figure S13. $^{31}P\{^{1}H\}$ NMR spectrum (162 MHz, DMSO-d_6) of compound (*Z*)-**1**·PF_6 measured at 295 K. Note: Due to the low sample concentration, the two outermost peaks of the septet signal are very weak.

HRMS spectra of title compounds

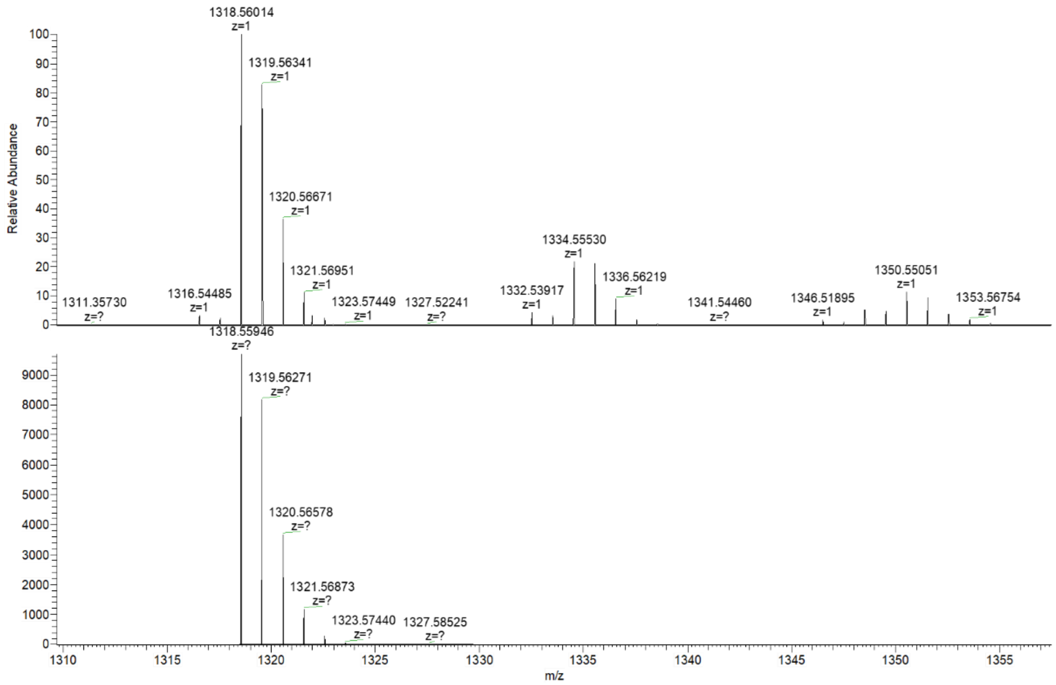

Figure S14. HRMS (ESI+) spectrum of (*Z*)-**1**·Cl showing the peak consistent with the positively charged interlocked rings without the chloride counterion (top) together with a simulated spectrum of the corresponding $C_{76}H_{80}N_5O_{16}^+$ fragment for reference (bottom).

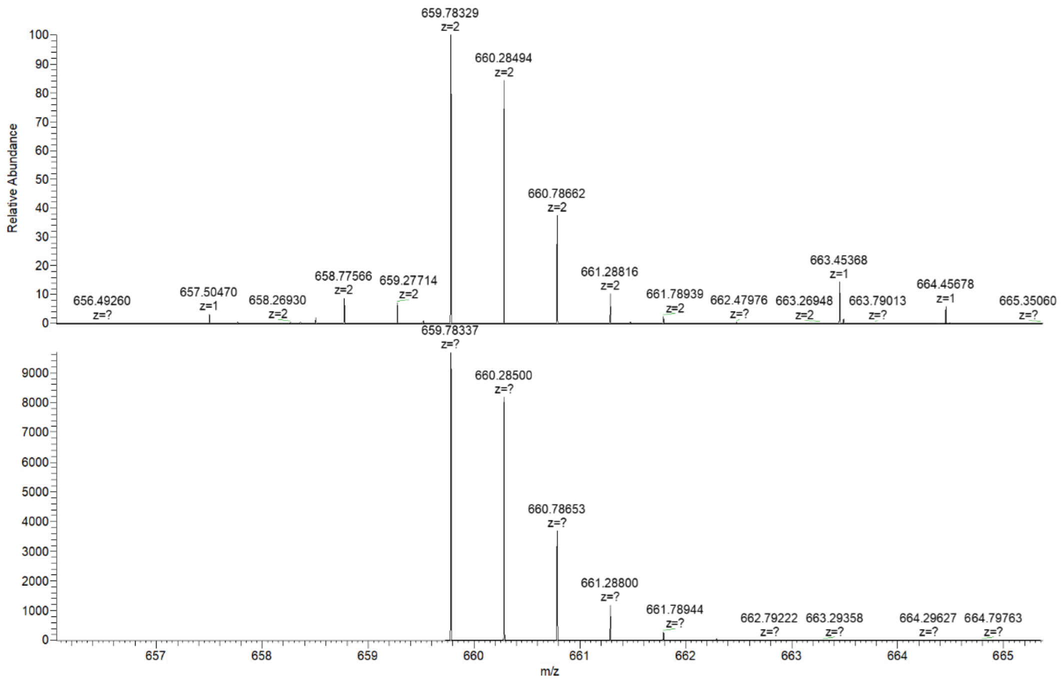

Figure S15. HRMS (ESI+) spectrum of (*Z*)-**1**·Cl showing the peak consistent with the doubly positively charged protonated interlocked rings without the chloride counterion (top) together with a simulated spectrum of the corresponding $C_{76}H_{81}N_5O_{16}^{2+}$ fragment for reference (bottom).

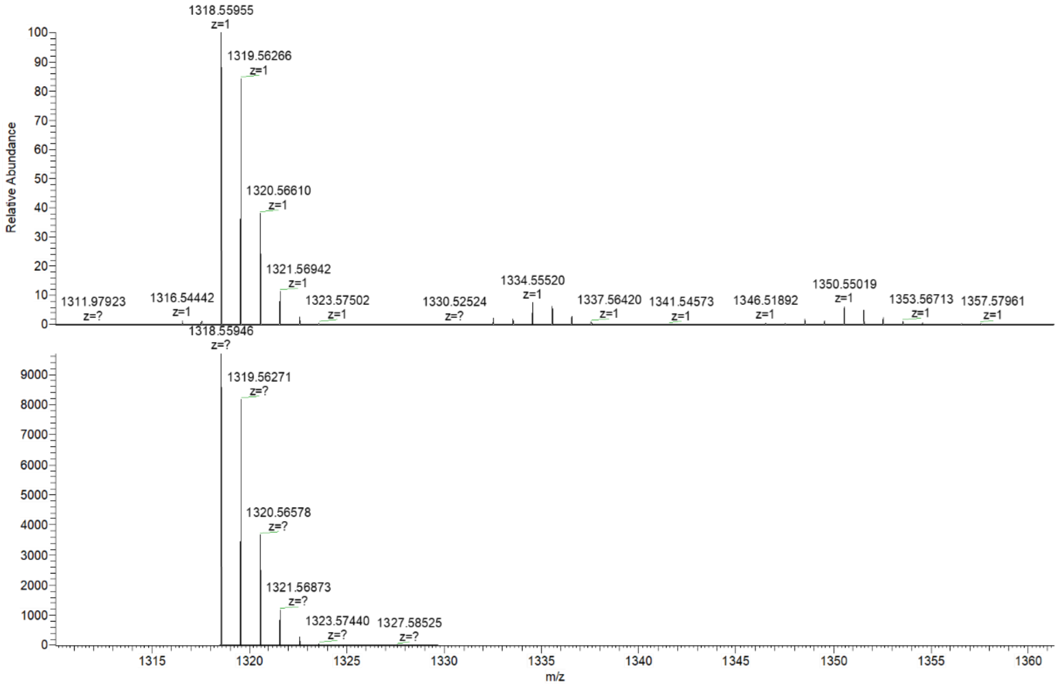

Figure S16. HRMS (ESI+) spectrum of (*Z*)-**1**·PF_6 showing the peak consistent with the positively charged interlocked rings without the hexafluorophosphate counterion (top) together with a simulated spectrum of the corresponding $C_{76}H_{80}N_5O_{16}^+$ fragment for reference (bottom).

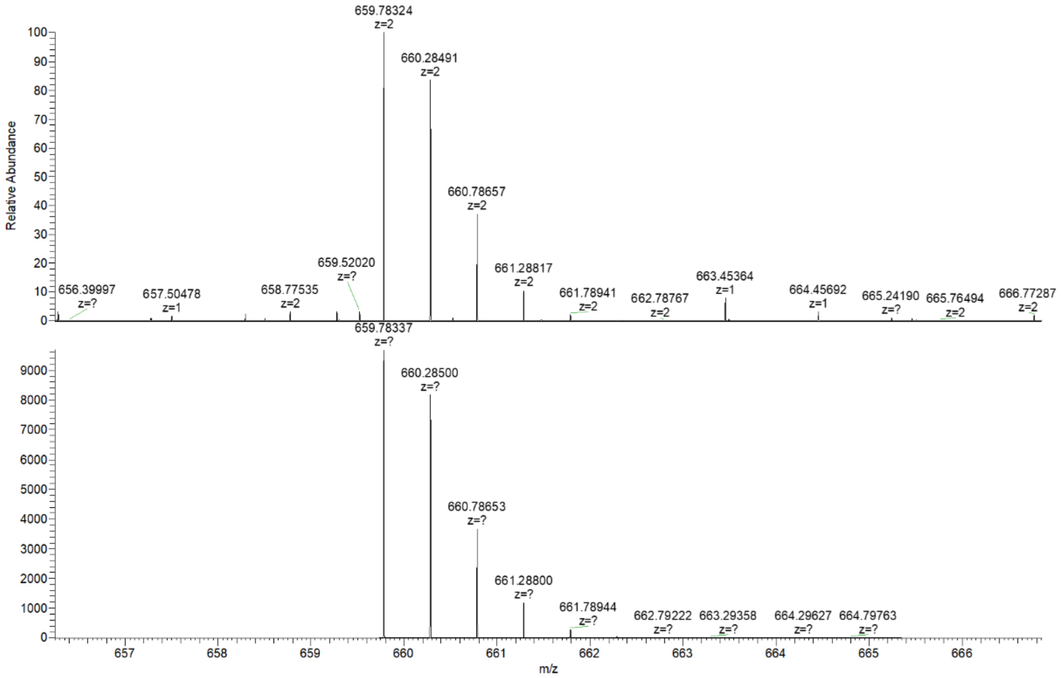

Figure S17. HRMS (ESI+) spectrum of (*Z*)-**1**·PF_6 showing the peak consistent with the doubly positively charged protonated interlocked rings without the hexafluorophosphate counterion (top) together with a simulated spectrum of the corresponding $C_{76}H_{81}N_5O_{16}^{2+}$ fragment for reference (bottom).

UV-Vis irradiation experiments

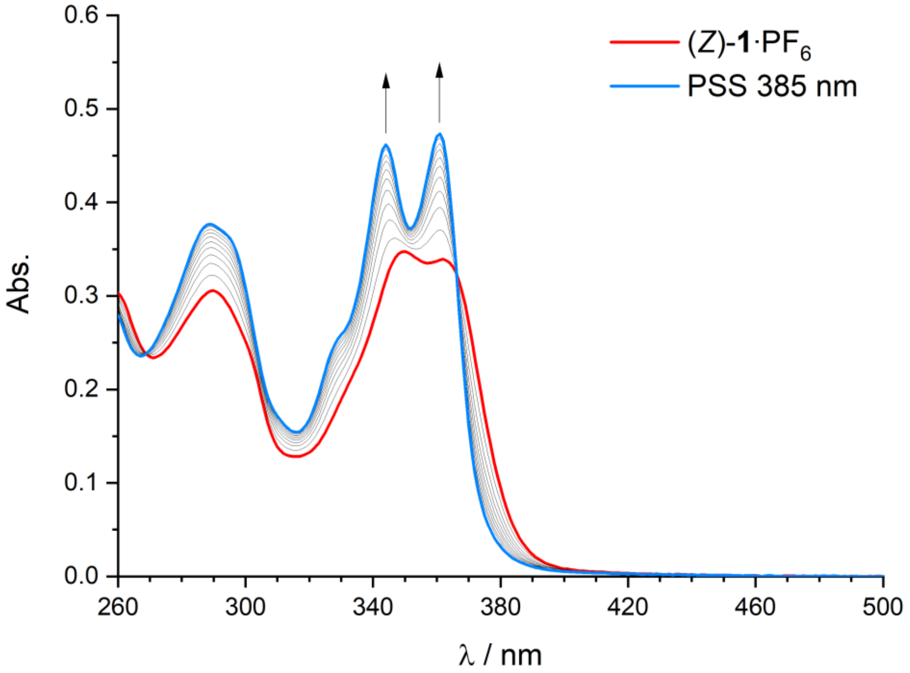

Figure S18. UV-Vis spectral changes of (*Z*)-**1**·PF_6 (2.0×10^{-5} M in dry and degassed DMSO) upon irradiation with 385 nm light for 50 s.

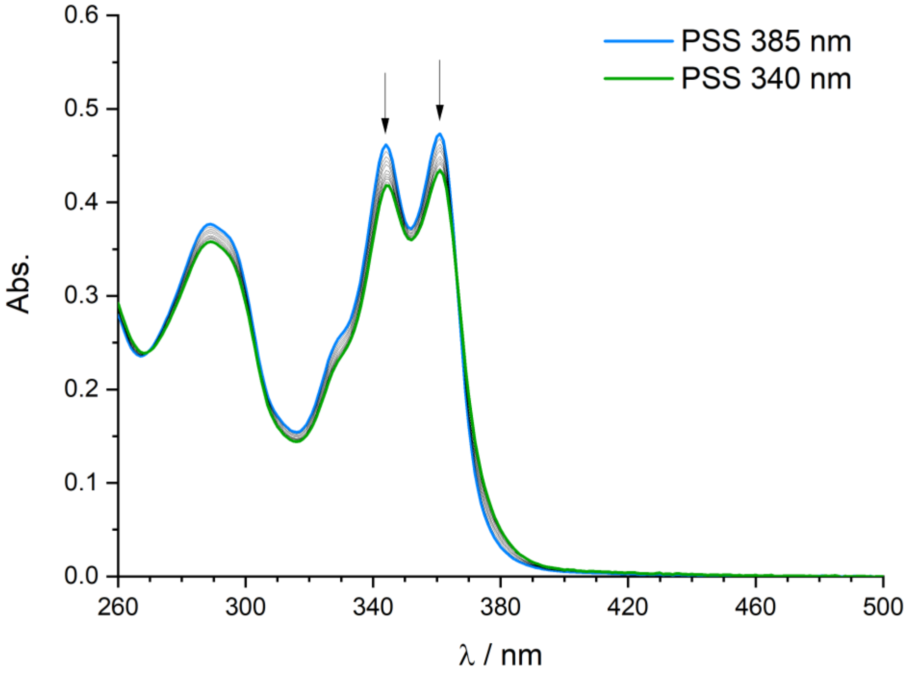

Figure S19. UV-Vis spectral changes of the PSS_{385} mixture of (*Z*)-**1**·PF_6 (2.0×10^{-5} M in dry and degassed DMSO) upon irradiation with 340 nm light for 120 s.

^{1}H NMR irradiation experiments

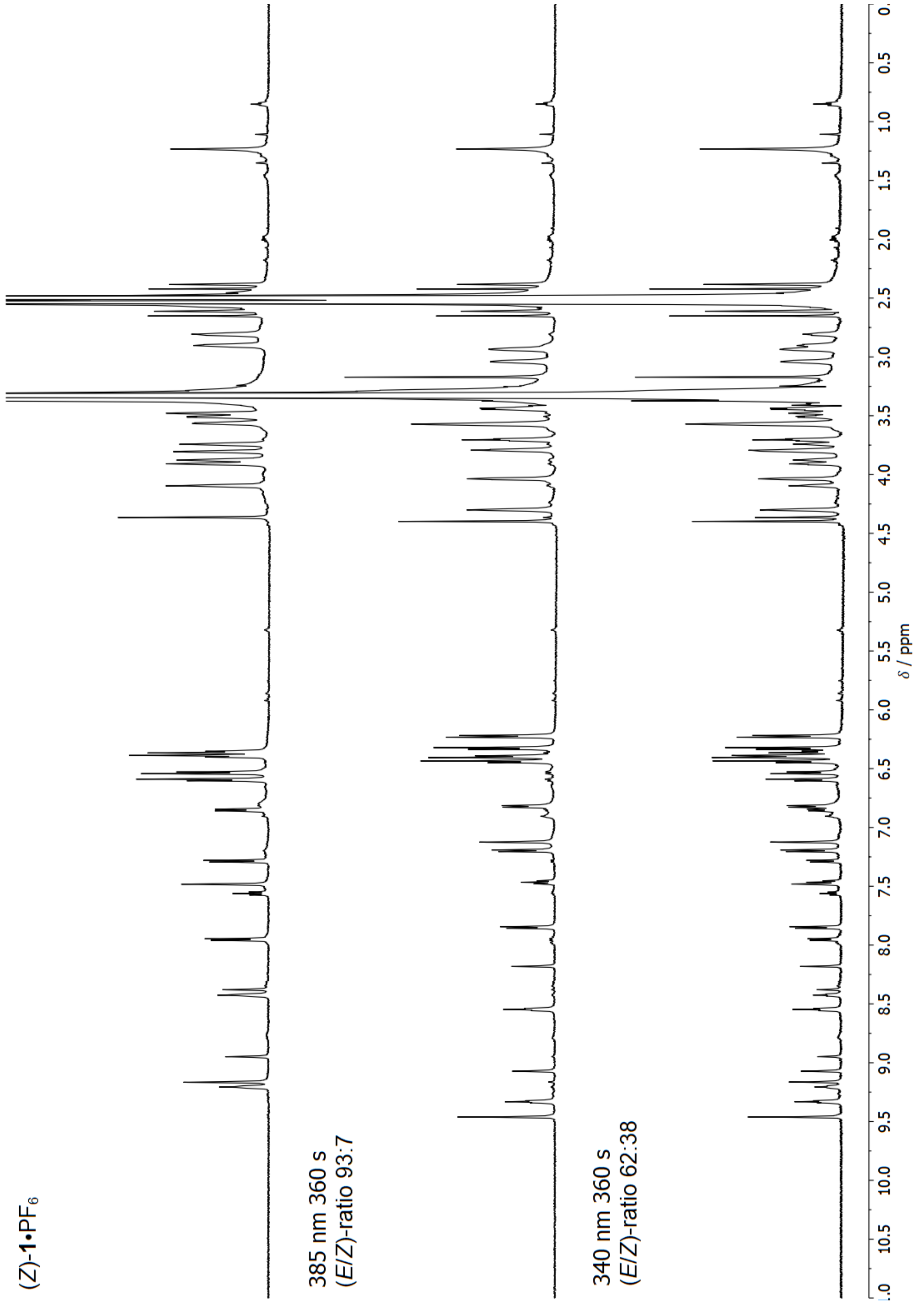

Figure S20. ^{1}H NMR spectral changes (600 MHz, 298 K) of (*Z*)-**1**·PF_6 (0.85 mM in degassed DMSO-d_6) upon irradiation with 385 nm light for 360 s, followed by 340 nm light for 360 s.

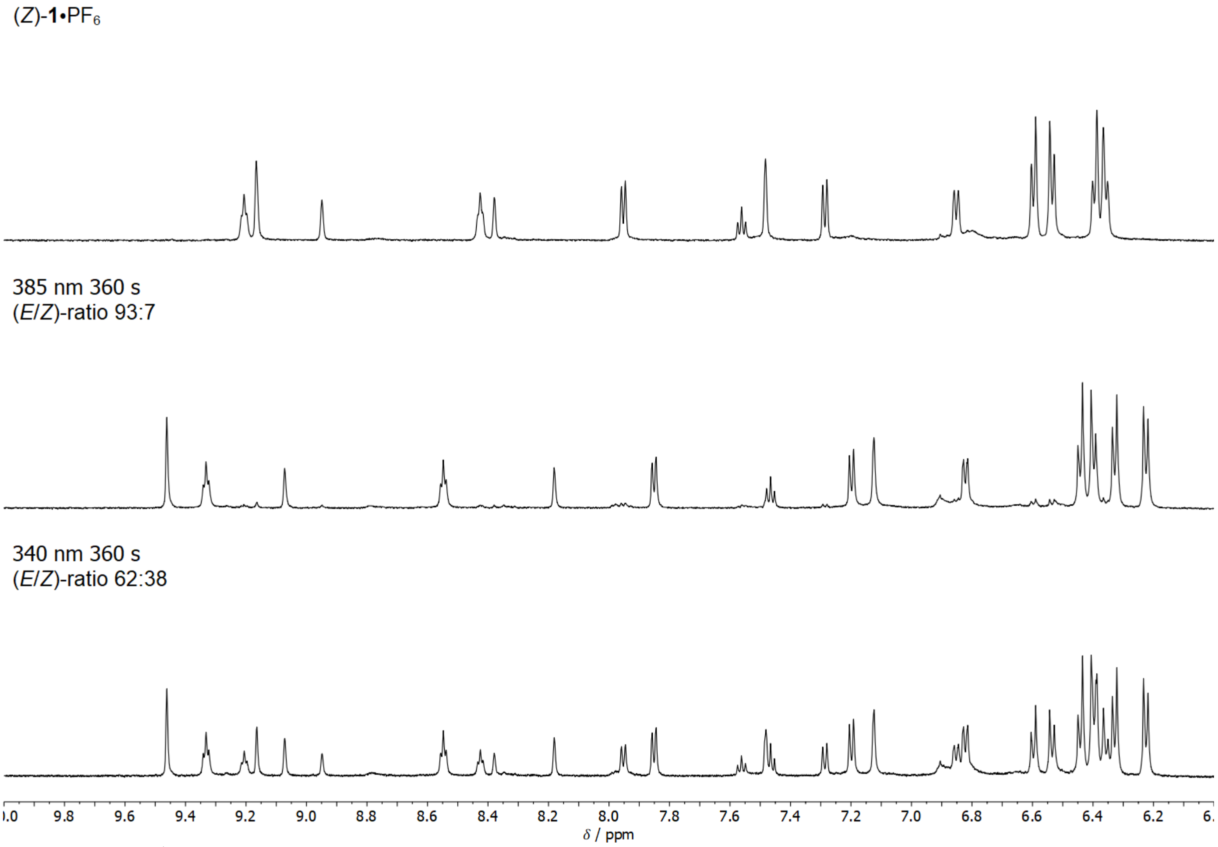

Figure S21. ^{1}H NMR spectral changes (600 MHz, 298 K) in the downfield region of (*Z*)-**1**·PF_6 (0.85 mM in DMSO-d_6) upon irradiation with 385 nm light for 360 s, followed by 340 nm light for 360 s.

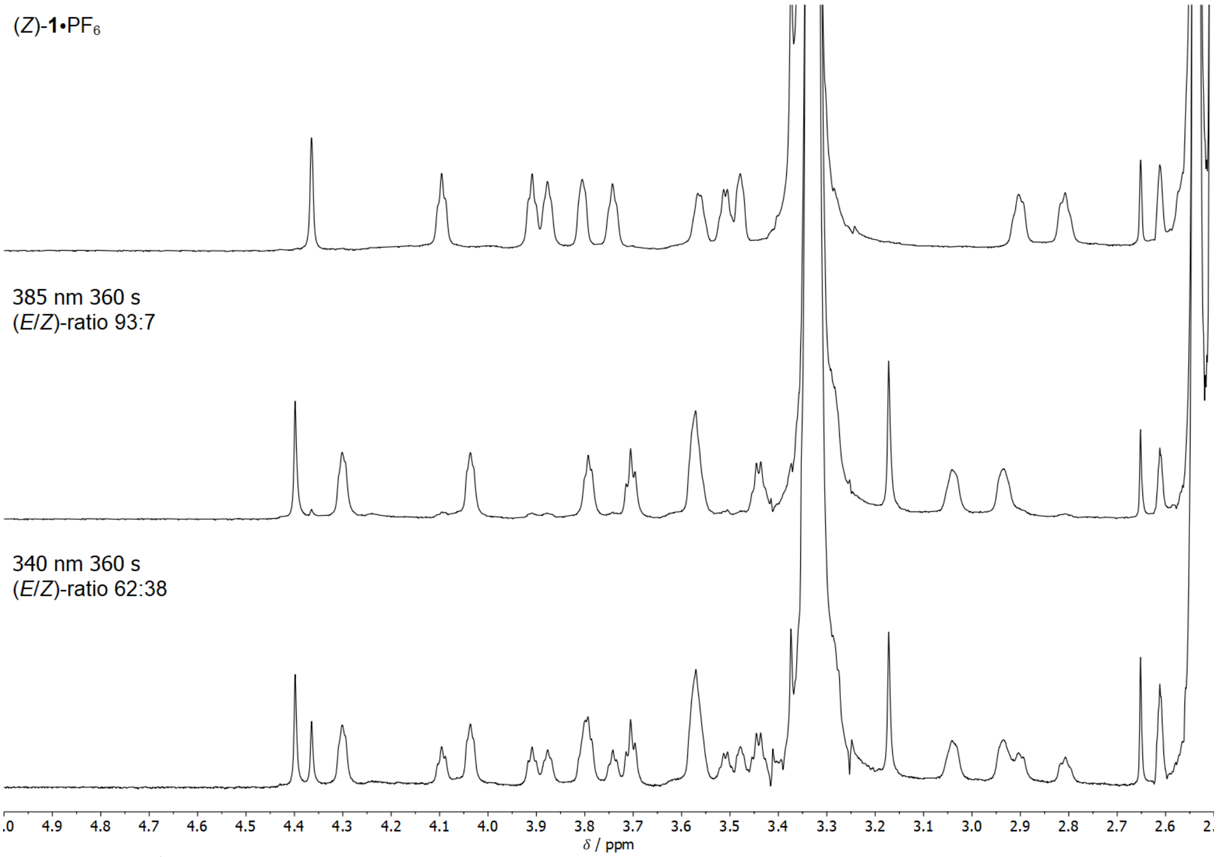

Figure S22. ^{1}H NMR spectral changes (600 MHz, 298 K) in the upfield region of (*Z*)-**1**·PF_6 (0.85 mM in DMSO-d_6) upon irradiation with 385 nm light for 360 s, followed by 340 nm light for 360 s.

^{1}H NMR titration experiments

For the titration experiments with (*Z*)-**1**·PF_6, solutions were prepared in either 1 mL DMSO-d_6 at 0.20 mM or in $CDCl_3$/CD_3OD (1:1, v/v) at 0.50 mM. Of these solutions, 0.5 mL was used to dissolve tetrabutylammonium chloride (NBu_4Cl), and this guest solution was added stepwise to 0.5 mL of the host solution. A ^{1}H NMR spectrum (600 MHz, 298 K) was recorded after each addition.

For the titration experiments with macrocycle (*E*)-**1**·PF_6 in DMSO-d_6, a 0.20 mM solution of the corresponding (*Z*)-isomer was prepared in 1 mL degassed DMSO-d_6. The solution was irradiated with 385 nm light for 2 min (i.e., until the photostationary state was reached). For the titration experiments with macrocycle (*E*)-**1**·PF_6 in $CDCl_3$/CD_3OD (1:1, v/v), a 0.50 mM solution of the corresponding (*Z*)-isomer was prepared in 1 mL $CDCl_3$/CD_3OD (1:1, v/v), where both solvents were first purged separately with argon for 10 min. The solution was irradiated with 385 nm light over the course of 6 min, at which time an (*E*/*Z*)-ratio of 82:18 was reached, as calculated by integration of the ^{1}H NMR signals of both isomers. Of these solutions containing (*E*)-**1**·PF_6, 0.5 mL was used to dissolve NBu_4Cl and this guest solution was added stepwise to 0.5 mL of the host solution, and a ^{1}H NMR spectrum (600 MHz, 298 K) was recorded after each addition.

The ^{1}H NMR titration data was fitted to a 1: 1 binding model using HypNMR software.[3] The experiment with (*E*)-**1**·PF_6 in $CDCl_3$/CD_3OD (1:1, v/v) was treated as a competitive titration, since a significant amount of (*Z*)-**1**·PF_6 (18%) was present after irradiation with 385 nm light. Therefore, the concentrations of both isomers were taken into account, and the association constant of the binding between (*Z*)-**1**·PF_6 and Cl^- in $CDCl_3$/CD_3OD (1:1, v/v), as derived from its independent titration experiment, was included during the fitting procedure.

In all cases, errors are estimated to be no more than ±15%.

Addition of NBu_4Cl to (*Z*)-1·PF_6 in DMSO-d_6:

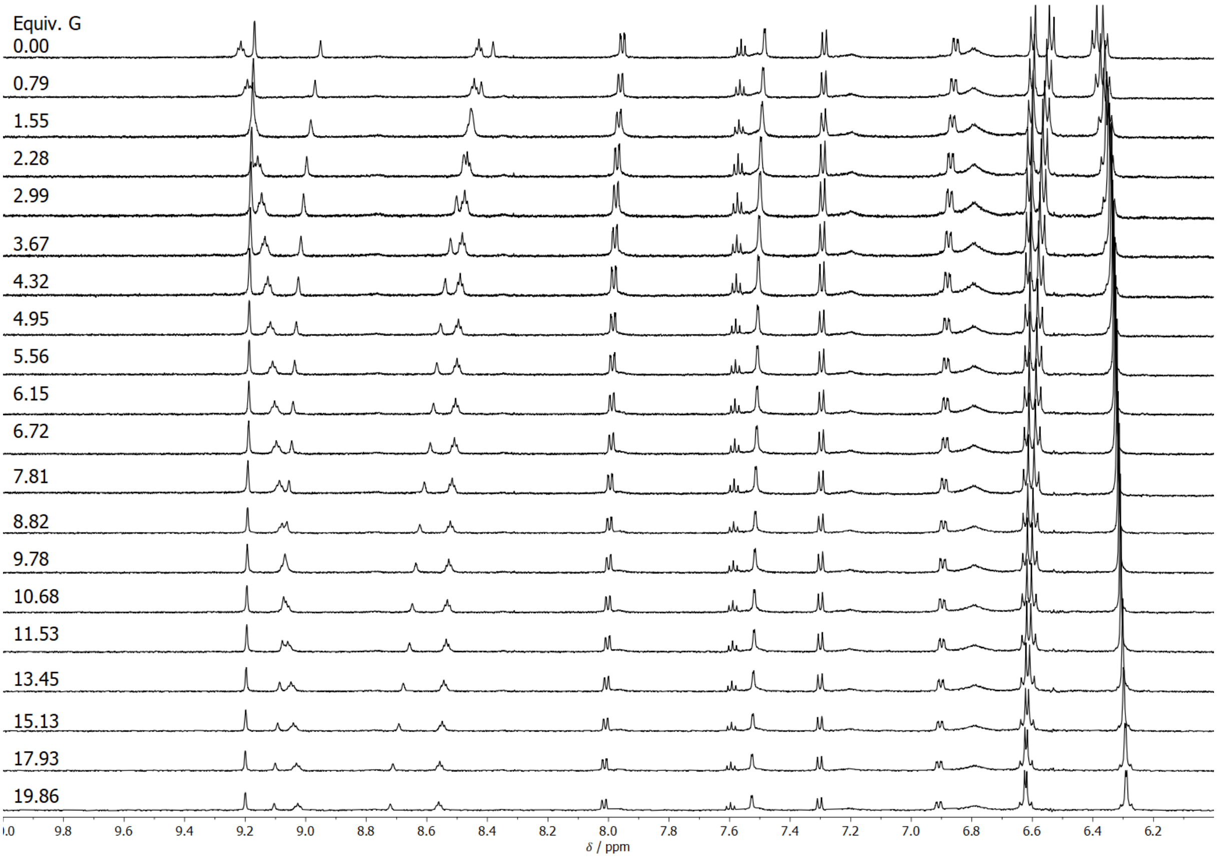

Figure S23. ^{1}H NMR spectral changes (600 MHz, 298 K) in the downfield region of (*Z*)-**1**·PF_6 (0.20 mM in DMSO-d_6) upon the stepwise addition of a 8.0 mM solution of NBu_4Cl.

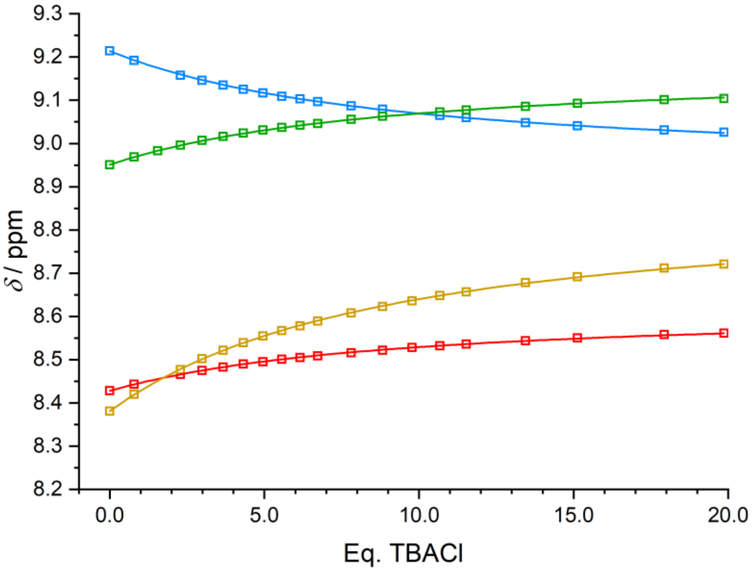

Figure S24. Chemical shift of the pyridinium protons N-$H_{d'}$ (blue) and C-$H_{c'}$ (green), as well as isophthalamide protons N-H_l (red) and C-H_m (yellow) during the titration and curve fitting obtained by using a 1:1 binding model with HypNMR software; $K_a = 6.0 \times 10^2$ M^{-1}.

Addition of NBu_4Cl to (E)-1·PF_6 in DMSO-d_6:

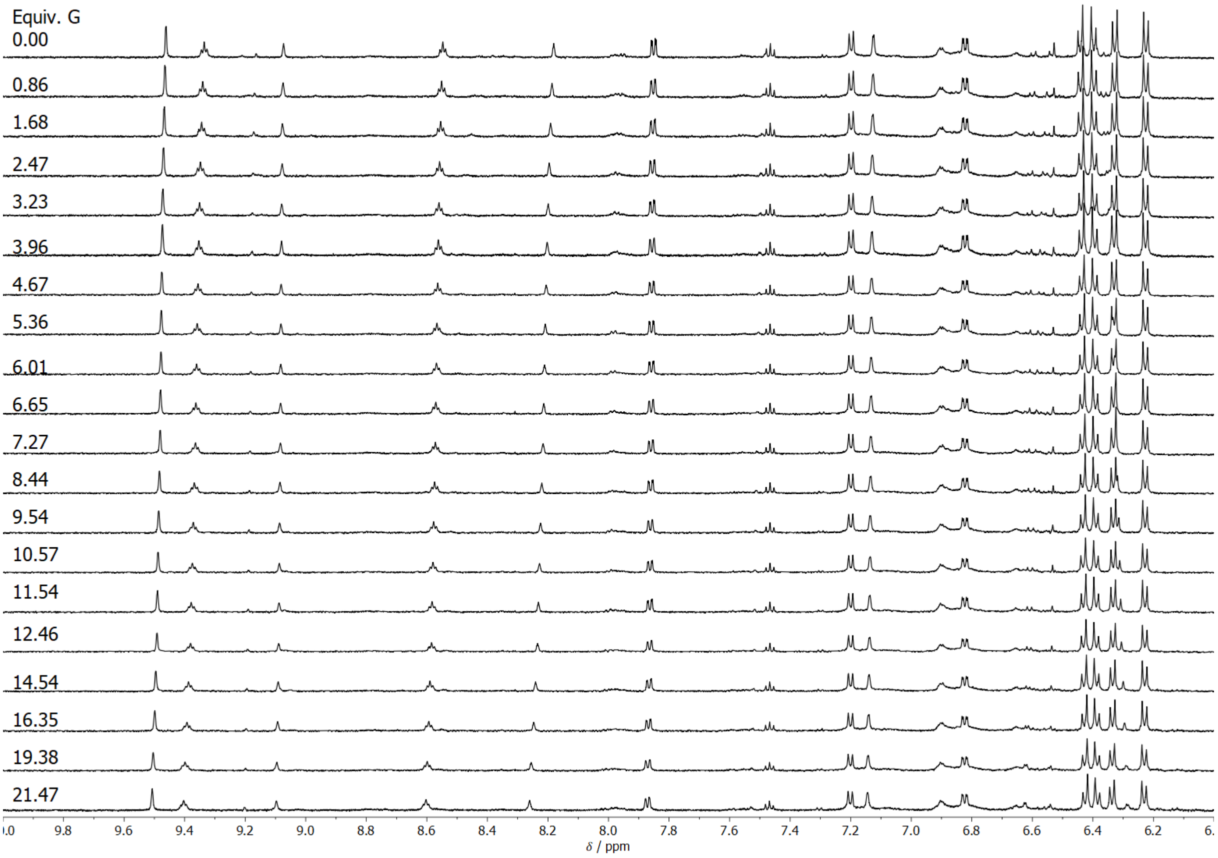

Figure S25. ^{1}H NMR spectral changes (600 MHz, 298 K) in the downfield region of (E)-**1**·PF_6 (0.20 mM in DMSO-d_6) upon the stepwise addition of a 8.6 mM solution of NBu_4Cl.

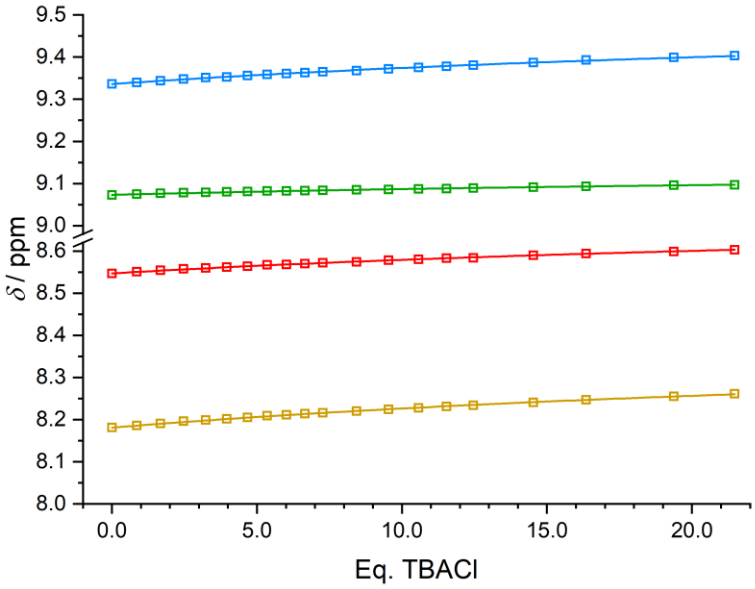

Figure S26. Chemical shift of the pyridinium protons N-$H_{d'}$ (blue) and C-$H_{c'}$ (green), as well as isophthalamide protons N-H_l (red) and C-H_m (yellow) during the titration and curve fitting obtained by using a 1:1 binding model with HypNMR software; $K_a = 1.3 \times 10^2$ M^{-1}.

Addition of NBu_4Cl to (*Z*)-1·PF_6 in $CDCl_3/CD_3OD$ (1:1, v/v):

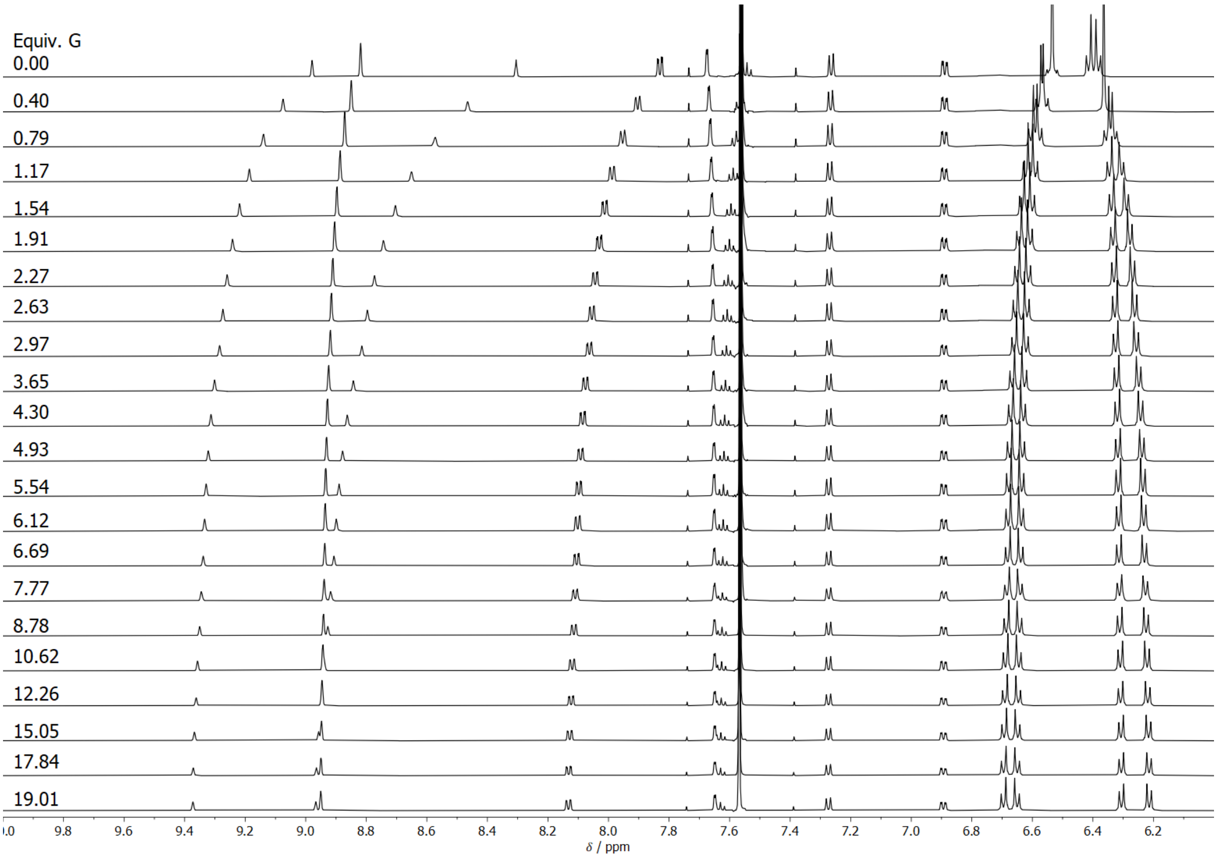

Figure S27. ^{1}H NMR spectral changes (600 MHz, 298 K) in the downfield region of (*Z*)-**1**·PF_6 (0.50 mM in $CDCl_3/CD_3OD$ [1:1, v/v]) upon the stepwise addition of a 20 mM solution of NBu_4Cl.

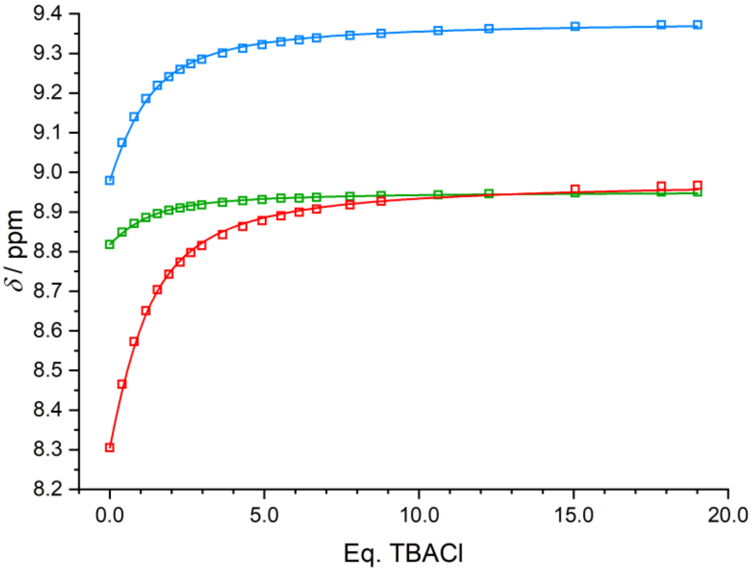

Figure S28. Chemical shift of the pyridinium protons C-H$_{c'}$ (blue) and C-H$_{b'}$ (green), as well as isophthalamide proton C-H$_m$ (red) during the titration and curve fitting obtained by using a 1:1 binding model with HypNMR software; $K_a = 3.0 \times 10^3$ M^{-1}.

Generation of (*E*)-1·PF_6 in $CDCl_3/CD_3OD$ (1:1, v/v):

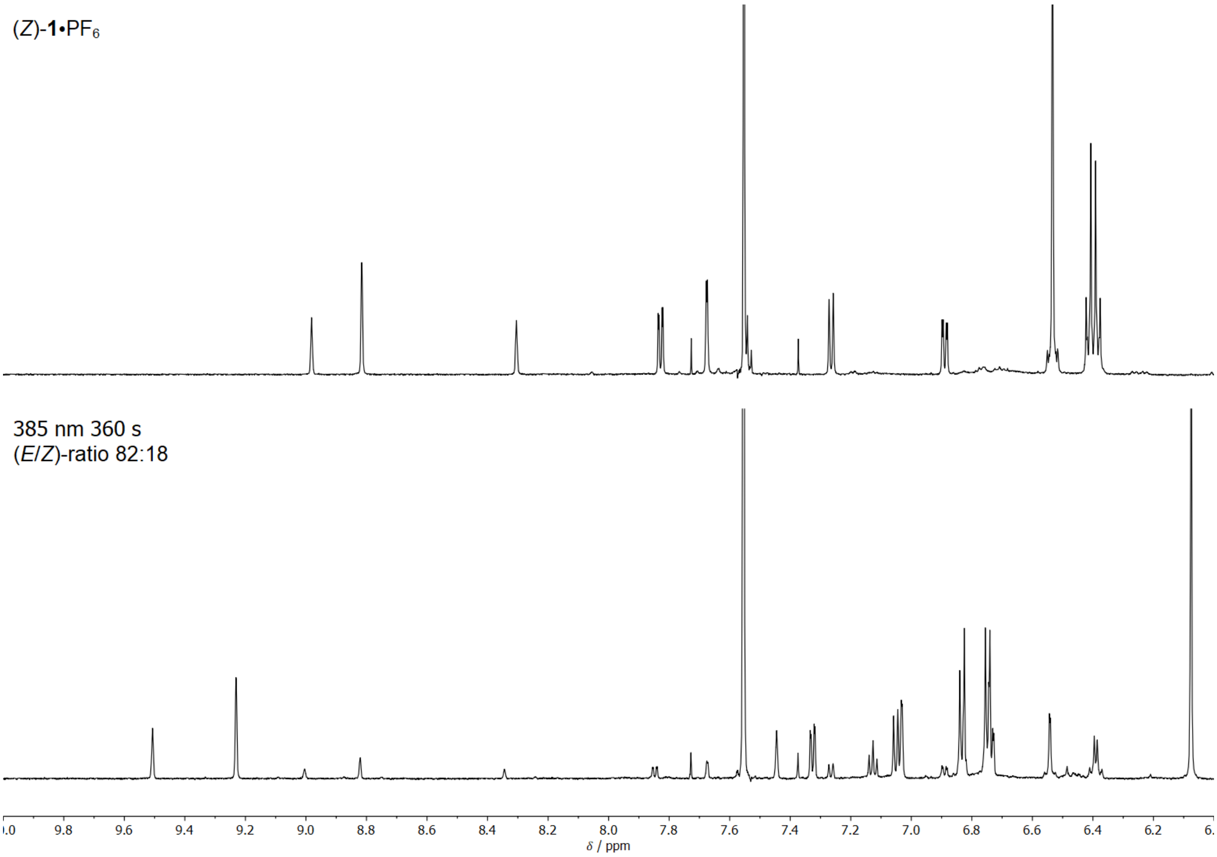

Figure S29. ^1H NMR spectral changes (600 MHz, 298 K) of (*Z*)-**1**·PF_6 (0.50 mM in $CDCl_3/CD_3OD$ [1:1, v/v]) upon irradiation with 385 nm light for 360 s, resulting in an *E*/*Z*-mixture with a ratio of 82:18.

Addition of NBu_4Cl to (*E*)-1·PF_6 in $CDCl_3/CD_3OD$ (1:1, v/v):

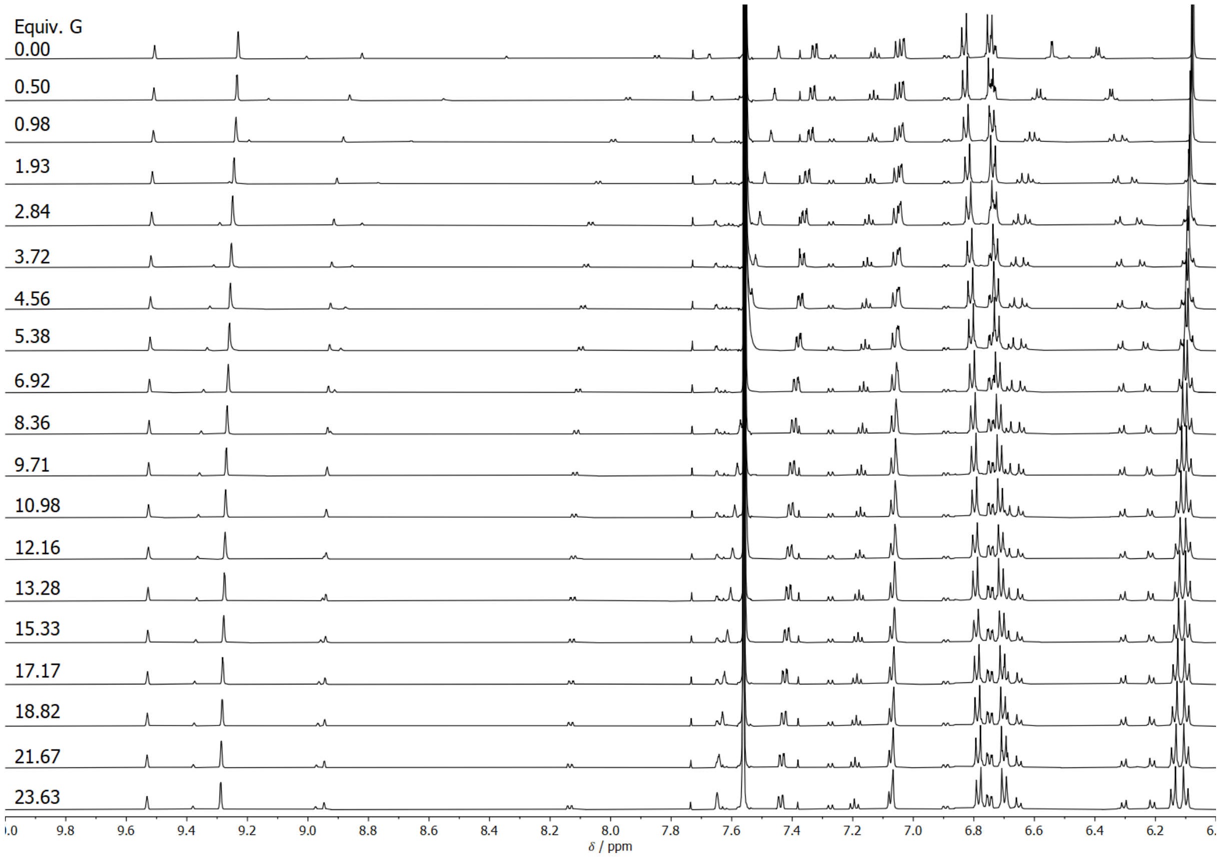

Figure S30. ^{1}H NMR spectral changes (600 MHz, 298 K) in the downfield region of (*E*)-**1**·PF_6 (0.50 mM in $CDCl_3/CD_3OD$ [1:1, v/v]) upon the stepwise addition of a 21 mM solution of NBu_4Cl.

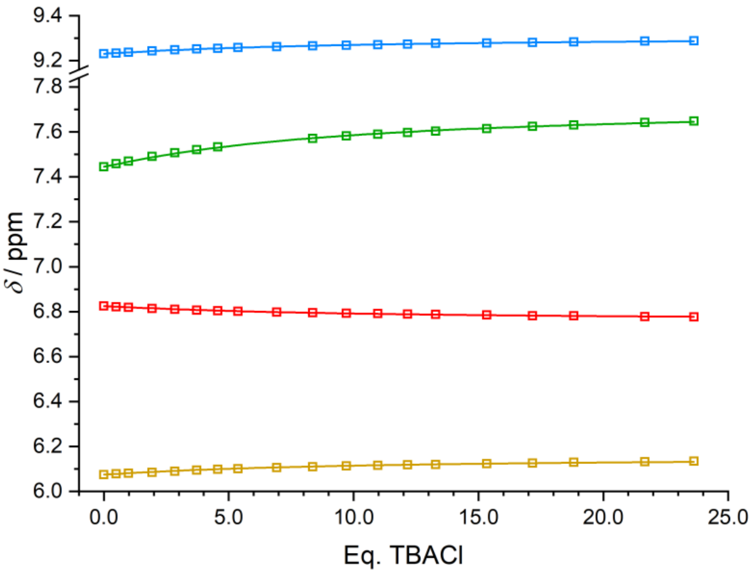

Figure S31. Chemical shift of the pyridinium proton C-$H_{b'}$ (blue), isophthalamide proton C-H_m (green) and two hydroquinone protons (red, yellow) during the titration and curve fitting obtained by using a 1:1 binding model with HypNMR software; $K_a = 2.6 \times 10^2$ M^{-1}.

^{1}H NMR irradiation in presence of chloride

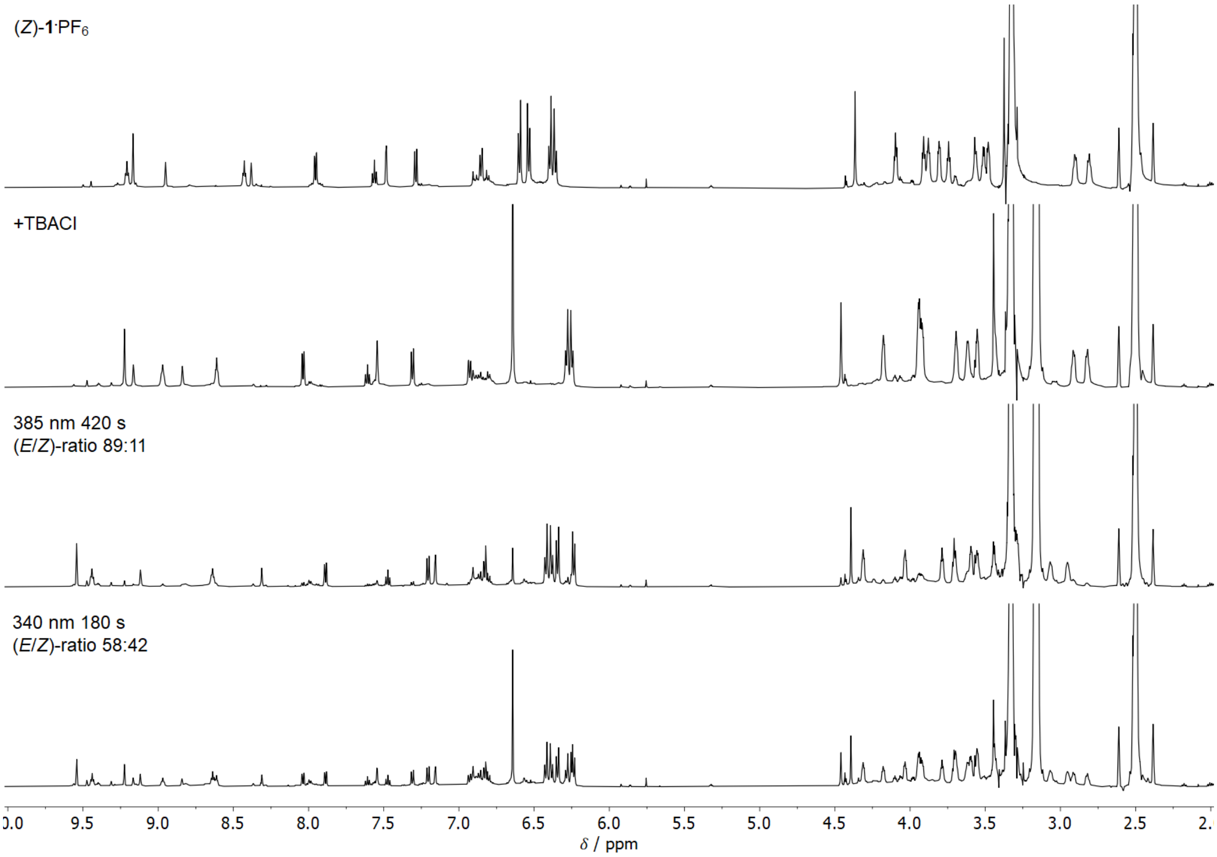

Figure S32. ^{1}H NMR spectral changes (600 MHz, 298 K) of a mixture of (*Z*)-**1**·PF_6 (1.0 mM in degassed DMSO-d_6) after addition of 15 equivalents of NBu_4Cl, followed by sequential irradiation with 385 nm light for 420 s and 340 nm light for 180 s.

The percentage of bound host was calculated using the observed chemical shifts of protons located in the binding site of (*Z*)-**1** and (*E*)-**1** ($H_{c'}$, $H_{d'}$, H_l and H_m) during the experiment, relative to those of the respective unbound hosts and their fully bound chloride complexes, which were derived from the ^{1}H NMR titrations in DMSO-d_6. The following formula was used to calculate the percentage:

$$\frac{\delta_{observed} - \delta_{unbound}}{\delta_{complex} - \delta_{unbound}} \times 100\%$$

The relevant chemical shifts are reported in the table below.

Table S1. Chemical shifts of protons $H_{c'}$, $H_{d'}$, H_l and H_m for the unbound forms and chloride complexes of (*Z*)-**1** and (*E*)-**1**, as well as their observed shifts during the irradiation experiment in the presence of 15 equivalents of NBu_4Cl.

	(*Z*)-**1**			(*E*)-**1**		
	$\delta_{unbound}$ (ppm)	$\delta_{complex}$ (ppm)	$\delta_{observed}$ (ppm)	$\delta_{unbound}$ (ppm)	$\delta_{complex}$ (ppm)	$\delta_{observed}$ (ppm)
$H_{c'}$	8.95	9.17	9.16	9.07	9.14	9.12
$H_{d'}$	9.21	8.94	8.97	9.34	9.52	9.44
H_l	8.43	8.62	8.61	8.55	8.70	8.64
H_m	8.38	8.87	8.84	8.18	8.40	8.31

Single crystal X-ray crystallography

All reflection intensities were measured at 110(2) K using a SuperNova diffractometer (equipped with Atlas detector) with Cu $K\alpha$ radiation (λ = 1.54178 Å) under the program CrysAlisPro (Version CrysAlisPro 1.171.42.49, Rigaku OD, 2022). The same program was used to refine the cell dimensions and for data reduction. The structure was solved with the program SHELXS-2018/3 (Sheldrick, 2018) and was refined on F^2 with SHELXL-2018/3 (Sheldrick, 2018). Analytical numeric absorption correction using a multifaceted crystal model was applied using CrysAlisPro. The temperature of the data collection was controlled using the system Cryojet (manufactured by Oxford Instruments). The H atoms were placed at calculated positions (unless otherwise specified) using the instructions AFIX 23, AFIX 43 or AFIX 137 with isotropic displacement parameters having values 1.2 or 1.5 U_{eq} of the attached C atoms. The H atoms attached to N23, N34, N63 and N92 were found from difference Fourier maps, and their coordinates were refined pseudofreely using the DFIX instruction in order to keep the N–H distances within an acceptable range.

Specified hydrogen bonds (with esds except fixed and riding H)

D-H	H...A	D...A	<(DHA)	
0.93(2)	2.50(2)	3.408(2)	166(3)	N23-H23...Cl1
0.88(2)	2.55(2)	3.410(2)	165(3)	N34-H34...Cl1
0.88(2)	2.57(2)	3.414(2)	164(2)	N63-H63...Cl1
0.88(2)	2.60(2)	3.4698(19)	170(2)	N92-H92...Cl1
0.95	2.76	3.622(3)	151.4	C31-H31...Cl1
0.95	2.57	3.519(2)	173.2	C96-H96...Cl1

The asymmetric contains one formula unit of the target compound and some amount of lattice solvent molecules. The moiety O66→O82 is disordered over two orientations, and the occupancy factor of the major component refines to 0.678(3). The asymmetric unit contains one partially lattice DCM molecule that is disordered over two orientations, and the occupancy factors of the major and minor components refine to 0.623(3) and 0.171(3), respectively. The crystal lattice also contains some amount of very disordered (and potentially partially occupied) lattice solvent molecule (likely to be MeCN), and their contribution was removed from the final refinement using the SQUEEZE procedure in Platon (Spek, 2009).[4]

Table S2. Crystallographic data for the structure of (*Z*)-**1**·Cl.

Crystal data	
Chemical formula	$C_{30}H_{36}N_3O_8 \cdot C_{46}H_{44}N_2O_8 \cdot 0.794(CH_2Cl_2) \cdot Cl$
M_r	1422.23
Crystal system, space group	Triclinic, *P*1
Temperature (K)	110
a, *b*, *c* (Å)	14.9514 (4), 16.3606 (7), 17.5556 (7)
α, β, γ (°)	80.287 (3), 65.353 (3), 69.019 (3)
V (Å^3)	3643.4 (3)
Z	2
Radiation type	Cu *K*α
μ (mm^{-1})	1.58
Crystal size (mm)	0.33 × 0.05 × 0.04
Data collection	
Diffractometer	SuperNova, Dual, Cu at zero, Atlas
Absorption correction	Analytical<br>*CrysAlis PRO* 1.171.42.49 (Rigaku Oxford Diffraction, 2022) Analytical numeric absorption correction using a multifaceted crystal model based on expressions derived by R.C. Clark & J.S. Reid. (Clark, R. C. & Reid, J. S. (1995). Acta Cryst. A51, 887-897) Empirical absorption correction using spherical harmonics, implemented in SCALE3 ABSPACK scaling algorithm.
T_{min}, T_{max}	0.774, 0.954
No. of measured, independent and observed [$I > 2\sigma(I)$] reflections	48041, 13000, 9314
R_{int}	0.045
$(\sin \theta/\lambda)_{max}$ (Å^{-1})	0.598
Refinement	
$R[F^2 > 2\sigma(F^2)]$, $wR(F^2)$, S	0.050, 0.147, 1.03
No. of reflections	13000
No. of parameters	1089
No. of restraints	652
H-atom treatment	H atoms treated by a mixture of independent and constrained refinement
$\Delta\rho_{max}$, $\Delta\rho_{min}$ (e Å^{-3})	0.63, -0.46

Computer programs: *CrysAlis PRO* 1.171.42.49 (Rigaku OD, 2022), *SHELXS2018*/3 (Sheldrick, 2018), *SHELXL2018*/3 (Sheldrick, 2018), *SHELXTL* v6.10 (Sheldrick, 2008).[5]

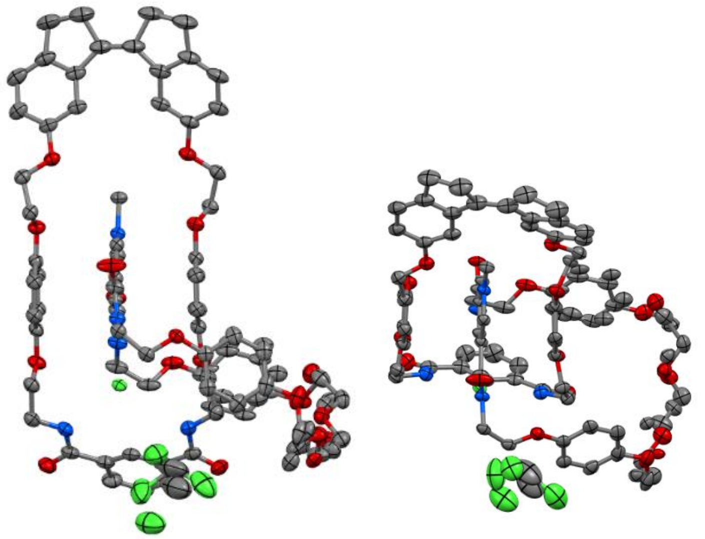

Figure S33. Displacement ellipsoid plot (50% probability level) of (*Z*)-**1**·Cl found in the crystal structure at 110(2) K. Hydrogen atoms and disorder in the oligo-ethylene glycol part of the pyridinium-containing macrocycle have been omitted for clarity.

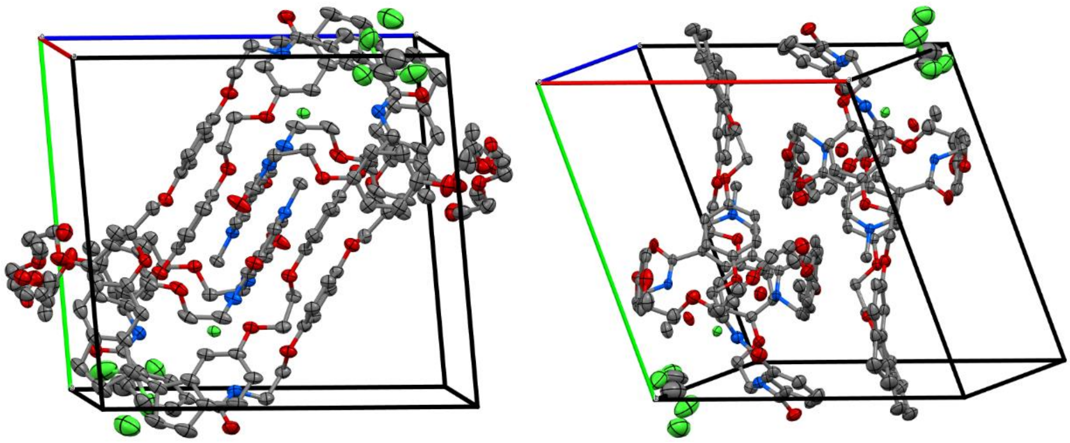

Figure S34. Crystal packing diagram of (*Z*)-**1**·Cl found in the solid state structure. Hydrogen atoms have been omitted for clarity. Both (*P*)- and (*M*)-isomers of (*Z*)-**1**·Cl were found in the solid-state structure (the structure is centrosymmetric), as well as some amount of lattice solvent molecules.

References

[1] J. de Jong, M. A. Siegler and S. J. Wezenberg, *Angew. Chem. Int. Ed.*, 2024, **63**, e202316628.

[2] L. M. Hancock, L. C. Gilday, N. L. Kilah, C. J. Serpell and P. D. Beer, *Chem. Commun.*, 2011, **47**, 1725–1727.

[3] C. Frassineti, S. Ghelli, P. Gans, A. Sabatini, M. S. Moruzzi and A. Vacca, *Anal. Biochem.*, 1995, **231**, 374–382.

[4] A. L. Spek, *Acta Crystallogr. Sect. D*, 2009, **65**, 148–155.

[5] G. M. Sheldrick, *Acta Crystallogr. Sect. C*, 2015, **71**, 3–8.
